# *In silico* analysis of crustacean hyperglycemic hormone family G protein-coupled receptor candidates

**DOI:** 10.3389/fendo.2023.1322800

**Published:** 2024-01-09

**Authors:** Mihika T. Kozma, Jorge L. Pérez-Moreno, Neha S. Gandhi, Luisanna Hernandez Jeppesen, David S. Durica, Tomer Ventura, Donald L. Mykles

**Affiliations:** ^1^ Department of Biology, Colorado State University, Fort Collins, CO, United States; ^2^ Department of Computer Science and Engineering, Manipal Institute of Technology, Manipal Academy of Higher Education, Manipal, Karnataka, India; ^3^ School of Chemistry and Physics, Queensland University of Technology, Brisbane, QLD, Australia; ^4^ Department of Biology, University of Oklahoma, Norman, OK, United States; ^5^ Centre for BioInnovation and School of Science, Technology and Engineering, University of the Sunshine Coast, Sippy Downs, QLD, Australia; ^6^ Coastal and Marine Sciences Institute, University of California-Davis Bodega Marine Laboratory, Bodega Bay, CA, United States

**Keywords:** molt-inhibiting hormone, crustacean hyperglycemic hormone, Y-organ, molting, ecdysteroid, neuropeptide, G protein-coupled receptor, CrusTome

## Abstract

Ecdysteroid molting hormone synthesis is directed by a pair of molting glands or Y-organs (YOs), and this synthesis is inhibited by molt-inhibiting hormone (MIH). MIH is a member of the crustacean hyperglycemic hormone (CHH) neuropeptide superfamily, which includes CHH and insect ion transport peptide (ITP). It is hypothesized that the MIH receptor is a Class A (Rhodopsin-like) G protein-coupled receptor (GPCR). The YO of the blackback land crab, *Gecarcinus lateralis*, expresses 49 Class A GPCRs, three of which (Gl-CHHR-A9, -A10, and -A12) were provisionally assigned as CHH-like receptors. CrusTome, a transcriptome database assembled from 189 crustaceans and 12 ecdysozoan outgroups, was used to deorphanize candidate MIH/CHH GPCRs, relying on sequence homology to three functionally characterized ITP receptors (BNGR-A2, BNGR-A24, and BNGR-A34) in the silk moth, *Bombyx mori*. Phylogenetic analysis and multiple sequence alignments across major taxonomic groups revealed extensive expansion and diversification of crustacean A2, A24, and A34 receptors, designated CHH Family Receptor Candidates (CFRCs). The A2 clade was divided into three subclades; A24 clade was divided into five subclades; and A34 was divided into six subclades. The subclades were distinguished by conserved motifs in extracellular loop (ECL) 2 and ECL3 in the ligand-binding region. Eleven of the 14 subclades occurred in decapod crustaceans. In *G. lateralis*, seven CFRC sequences, designated Gl-CFRC-A2α1, -A24α, -A24β1, -A24β2, -A34α2, -A34β1, and -A34β2, were identified; the three A34 sequences corresponded to Gl-GPCR-A12, -A9, and A10, respectively. ECL2 in all the CFRC sequences had a two-stranded β-sheet structure similar to human Class A GPCRs, whereas the ECL2 of decapod CFRC-A34β1/β2 had an additional two-stranded β-sheet. We hypothesize that this second β-sheet on ECL2 plays a role in MIH/CHH binding and activation, which will be investigated further with functional assays.

## Introduction

Molting processes in decapod crustaceans are controlled by ecdysteroids synthesized by a pair of molting glands, or Y-organs (YOs) (1–3). Molt-inhibiting hormone (MIH), released from the X-organ/sinus gland in the eyestalks, inhibits YO ecdysteroidogenesis through a cyclic nucleotide-dependent signaling pathway (4). In a proposed model, MIH binding to a high-affinity G protein-coupled receptor (GPCR) induces a cAMP/Ca^2+^-dependent triggering phase that leads to a prolonged NO/cGMP-dependent summation phase, which maintains the YO in the basal state between MIH pulses (2, 3, 5). It is hypothesized that activation of cGMP-dependent protein kinase leads to inhibition of mechanistic Target of Rapamycin Complex 1 (mTORC1)-dependent ecdysteroidogenesis (6). When conditions are suitable for molting, reduced MIH release activates the YO; rising hemolymph ecdysteroid titer drives the transition from the intermolt stage to the premolt stage (2, 3, 7).

MIH is a member of the crustacean hyperglycemic hormone (CHH) superfamily of neuropeptides. CHHs are characterized by six conserved cysteines that form three intramolecular disulfide bridges in the mature peptide (8, 9). They are classified into two types based on the transcript processing, precursor protein sequence, and post-translational modifications (8). Type I peptides (CHH and insect ion transport peptide or ITP) have an N-terminal signal peptide sequence, precursor-related peptide sequence, a KR cleavage site, and mature peptide (10, 11). Isoforms are generated by alternative mRNA splicing and chemical modifications of the N- and C-termini are common (5, 10, 11). Type II peptides (MIH, gonad-inhibiting hormone or GIH, and mandibular organ-inhibiting hormone or MOIH) lack the precursor-related peptide and KR cleavage site, having only the signal peptide and mature peptide sequences (3, 9). In addition, the N-terminal sequences of the Type II mature peptides have a conserved glycine (Gly12) inserted at the fifth position after the first cysteine (3). No isoforms are generated by alternative splicing in type II peptides and post-translational modifications are uncommon (3, 5). CHH superfamily mature peptides have a compact native conformation stabilized by the three disulfide bridges and nine conserved hydrophobic residues (3, 9, 12, 13). Type I peptides have four α-helices and type II peptides have the four α-helices and an additional short α1/3_10_-helix located around the conserved Gly12 (5, 9). Functional studies of expressed mutant MIH recombinant constructs show that both the N- and C-terminal regions, which come in close apposition in the native structures, contribute to MIH activity (9, 13, 14). Interestingly, the two residues at positions #13 and #14 in the α1 helix, but not the Gly12 itself, are critical for full MIH activity (9, 14).

The identity of the MIH receptor has eluded researchers for decades. Earlier efforts using covalent cross-linking of radiolabeled MIH with YO membrane proteins are inconclusive, as labelled proteins were neither identified nor characterized in a functional assay (15, 16). Transcriptomic analyses revealed that the YO expresses dozens of GPCRs, which are organized into six main classes, which include rhodopsin-like (Class A), secretin-like (Class B), and metabotropic glutamate/pheromone (Class C) designations (6). For example, the YO transcriptome of the blackback land crab, *Gecarcinus lateralis*, expresses 99 GPCRs: 49 in Class A, 35 in Class B, and 9 in Class C (17). More recent efforts have used *in silico* analysis of the growing number of crustacean transcriptome databases to identify CHH superfamily receptor candidates, taking advantage of homologies with insect ITP GPCRs (6). Functional analysis of silk moth (*Bombyx mori*) GPCRs identified two ITP receptors and one ITP/tachykinin receptor, designated *Bombyx* neuropeptide G-coupled receptor (BNGR)-A2 and -A34 and BNGR-A24, respectively (18). Using the BNGR sequences, Veenstra (19), identified four ITP GPCR homologs in the crayfish (*Procambarus clarkii*) neuropeptidome assembled from seven short read archives and three transcriptome shotgun assemblies (TSAs), including a TSA from the YO (20). Phylogenetic analysis showed that Pc-GPCR-A9 clustered with BNGR-A24 and Pc-GPCR-A52, -A53, and -A63 clustered with BNGR-A34 (19). Subsequently, putative CHH family receptors were identified in transcriptomes from green shore crab, *Carcinus maenas*; spiny lobsters, *Sagmariasus verreauxi* and *Panulirus argus*; blackback land crab, *Gecarcinus lateralis*; mud crab, *Scylla paramamosain*; swimming crab, *Portunus trituberculatus*; Chinese mitten crab, *Eriocheir sinensis*; American lobster, *Homarus americanus*; Norway lobster, *Nephrops norvegicus*; and blue crab, *Callinectes sapidus* (17, 21–25).

All GPCRs are single polypeptides with seven transmembrane α-helices (TMM1 to TMM7) connected by three extracellular loops (ECL1, 2, and 3) and three intracellular loops (ICL1, 2, and 3) (26–28). Vertebrate Class A GPCRs share conserved residues and motifs that have structural or activation functions (29, 30). ECL1, ECL2, and ECL3 have critical roles in ligand recognition and receptor function (26, 28, 31–35). In human Class A GPCRs, Y/HxWxF or xWxF motifs in the ECL1 interact with bound peptides (28, 34). The ECL2 is the most structurally diverse with specific amino acid residues that determine peptide binding affinity and specificity among receptor subfamilies (27, 28, 31, 32, 34). A disulfide bridge between a cysteine (C) at the extracellular surface of TMM3 and a cysteine (C) in the ECL2 β-sheet constrains the conformation of the seven transmembrane domain (27, 31, 32, 35). A CWxP motif in TMM6 is critical for receptor activation upon binding of a ligand (29, 36). A TxP motif in TMM2, conserved in chemokine receptors, and a NPxxY motif located at the intracellular surface of TMM7, are also involved in receptor activation (28, 29). An E/DRY motif at the TMM3/ICL2 boundary and amino acid residues in ICL3 interact with G proteins (27, 29, 37). Moreover, differences in amino acid sequences and lengths in the ICL3 region confer binding specificity to different G proteins (37, 38).

Any effort to identify the MIH receptor must start with a comprehensive search for ITP receptor homologs in crustacean transcriptomes, particularly those detected in the YO. Previous efforts using sequence homology, though partially successful, were hampered by fragmented and siloed databases, representing a relatively small number of species and taxonomic groups (17, 19, 21–25). CHH/MIH/GIH/MOIH peptides probably arose after the Hexapoda-Malacostraca split approximately 515 million years ago (39, 40). Therefore, it is likely that receptors to the CHH superfamily are ancient and that their lineage can be traced back to ecdysozoan ancestors in the Cambrian Period. Here we report the use of CrusTome, a multi-species, multi-tissue, transcriptome database of 201 assembled mRNA transcriptomes from 189 crustaceans and 12 ecdysozoan outgroups (41), to *in silico* deorphanize candidate CHH family Class A GPCRs, relying on sequence homology to the three *B. mori* ITP receptors. Putative homologs of BNGR-A2, BNGR-A24, and BNGR-A34 were identified in transcriptomes across Crustacea and annotated as CHH family receptor candidates (CFRCs). Among decapod crustaceans that have historically served as model organisms for molt regulation, seven CFRCs in *G. lateralis*, eight CFRCs in *P. clarkii*, and eight in *C. maenas* were identified. Multiple sequence alignments, phylogenetics, and molecular modeling of predicted receptor proteins identified structural features and conserved motifs in ECL2 and ECL3, which form the ligand-binding region. These features and motifs can be used to distinguish members of CFRC clades and subclades, and suggest mechanisms for ligand binding specificity. *In silico* modeling of Gl-MIH, Gl-CHH, and Gl-CFRC-A24 and -A34 protein structures was conducted, as *G. lateralis* is an established model for the study of molting physiology and endocrinology (2, 7, 42). Based on phylogeny, sequence analysis, and molecular modeling, we hypothesize that CFRC-A34β1 and CFRC-A34β2 are the MIH receptors in decapod crustaceans and should be prioritized for functional assays.

## Materials and methods

### Data sourcing

Transcriptomic datasets from the model crab species *G. lateralis* arising from previous work were obtained from public repositories and incorporated into the analyses. These datasets included transcriptomes for *G. lateralis* eyestalk ganglia (**Supplementary Data 1**) and YO under different experimental conditions (43–45).

### Sequence acquisition and curation

Reference sequences for *B. mori* A2, A24, and A34 ITP receptors were obtained from (18) (GenBank accessions NP_001127737.1, NP_001127722.1, NP_001127750.1, respectively). Other CFRC reference sequences were sourced from the NCBI GenBank database, with a particular focus on hexapod sequences that were classified as potential ITP receptor candidates, related GPCRs, and sequences that were previously identified from crustaceans (17, 19, 21–23). These sequences served as the input for iterative NCBI-BLAST (blastp 2.13.0+) searches with the intent of retrieving a sufficiently broad array of sequences for phylogenetic inference. Blastp searches were carried out against several databases: nr database of NCBI against taxon id “Crustacea,” CrusTome database (v.0.1.0) (41), and CrustyBase (46), as well as against previously published *G. lateralis* transcriptome assemblies (43–45).

Subject hits with e-value < e^-10^ were selected for further screening. The screening process consisted of the following: 1) Interproscan (version 5.64) analysis to determine if the BLAST hits were all class A GPCRs and contained the seven transmembrane domain region (IPR000276/PF00001: 7tm_1); 2) Any sequences that had less than six transmembrane regions as analyzed by TmHMM v2 were discarded; and 3) Redundant sequences from the same species were manually removed following evaluation of percent sequence homology following multiple sequence alignments and construction of maximum likelihood phylogenetic tree construction (see below). Exceptions were made in steps #1 and #2 if fragmented sequences were from brachyuran crabs. Hits were refined to retain only those that were complete or nearly complete sequences based on their length and domain regions with the aim to maximize the phylogenetic diversity and signal of the dataset, while preserving representation of focal clades, such as order Decapoda and the infraorder Brachyura (true crabs).

### Multiple sequence analyses and phylogenetics

The resulting putative CFRC sequences were aligned using the multiple sequence aligner, Multiple Alignment using Fast Fourier Transform (MAFFT; v.7.490 (47). Other subclasses of class A GPCRs from *G. lateralis*, which were previously annotated, served as outgroups (Proctolin, FMRF, Allatostatin, and HPR1 receptors) (17). The parameters for MAFFT alignment were chosen to prioritize accuracy over speed and to allow for large unaligned regions if encountered (“–dash –ep 0 –genafpair –maxiterate 10000” (47), thus fine-tuning the process for proteins that are typically challenging to align due to their particular structural characteristics (e.g., GPCRs (48). The –dash parameter equipped MAFFT with the capability to query a ‘Database of Aligned Structural Homologs,’ thereby integrating structural data to guide and optimize the alignment process (49). Subsequently, the generated alignment was trimmed using ClipKit in smart-gap mode (50), an alignment trimming tool proficient at discerning and preserving phylogenetically informative sites and facilitating more accurate phylogenetic inference. Maximum-likelihood phylogenies were reconstructed using IQ-TREE2 (51), applying a Jones-Taylor-Thornton amino acid replacement matrix under a FreeRate model with 9 rate categories [JTT+R9 (52–55)], as suggested by ModelFinder for the trimmed alignment (56). The phylogenetic tree derived from this initial reconstruction was subjected to TreeShrink for automated detection and removal of outliers/paralogs, setting the α value at 0.05 (57). The pruned alignment was then subjected to a second, final round of phylogenetic reconstruction using IQ-TREE2 (51) to enable confident characterization and annotation of the target proteins in a phylogenetic context. A subsequent IQ-TREE2 phylogenetic reconstruction was performed using identical model parameters as previously utilized [JTT+R9 (52–55)]. The branch support for this final phylogeny was assessed in a bipartite manner, using the Ultra-Fast Bootstrap approximation (UFBoot; 10,000 iterations) and an approximate Bayes test (58–60). The process was repeated independently for those sequences falling within the A24 and A34 clades to obtain better resolution of terminal branches in clade-specific phylogenies. Reference sequences and their corresponding Accession Identifiers are cataloged in **Supplementary Data S2**.

Multiple sequence alignments were produced for selected species across the phylogeny to compare the diversity of the ECL2 regions among the A2, A24, and A34 clades. These alignments were generated with the previously mentioned MAFFT strategy and subsequently visualized with a custom script to assess sequence content and conservation across clades and species (code available at: https://github.com/invertome/scripts/tree/main/plots). In addition, the script generates sequence logo plots depicting the proportion of each residue found per site in the alignment. Amino acid residue colors that are proximal in color space, in both the alignments and logo plots, denote similarities in physicochemical characteristics of the corresponding residues (61). Additionally, a deep-learning algorithm was employed to detect, predict, and annotate the topology of the candidate GPCRs (62) to delineate intracellular, extracellular, and transmembrane regions. Similarly, a subset of decapod species was selected to generate and plot multiple sequence alignments of the CHH and MIH peptides, as well as the A34 clade to further compare the sequences in ECL2, TMM5, ICL3, TMM6, and ECL3 regions.

### Protein structural modeling

Neural network-based methods AlphaFold and RoseTTAFold have outperformed homology modeling programs like Modeller for GPCR modeling in the absence of good templates (26, 63–65). Consequently, we used AlphaFold2 for the structural modeling of *G. lateralis* CFRC, Gl-MIH, and Gl-CHH sequences. The RoseTTAFold web service was used with default settings to predict the structures of each protein sequence, and the structural features were compared with the AlphaFold2 models.

UCSF ChimeraX version 1.5, a free multi-platform molecular modeling program developed by the Resource for Biocomputing, Visualization, and Informatics at the University of California, San Francisco, was used to model and visualize three-dimensional structures (66, 67). Full-length predicted protein of Gl-CFRC-A24α, -A24β1, -A34α2, -A34β1, and -A34β2 were selected for structural modeling; partial sequences of Gl-CFRC-A2α1 and -A24β2 were excluded. The sequences were truncated at the N- and C-termini due to intrinsic disorder of the N-terminal and C-terminal domains. The truncated sequences (**Supplementary Data 3**) were subjected to AlphaFold2 using the ChimeraX interface to submit three-dimensional structure prediction to run at Google Colab (68). The predicted structures were energy minimized and the best model out of five was selected for further analysis. AlphaFold2 has been used to predict either active or inactive states of GPCRs (69). However, the use of these sophisticated techniques was not feasible for this study, due to the lack of three-dimensional structures for crustacean GPCRs with their ligands. The RoseTTAFold web service was used with default settings to predict five structures of each protein sequence, and the structural features were compared with the AlphaFold2 models.

## Results

### Identification and classification of ITP GPCR homologs in crustaceans

**Figure 1:**
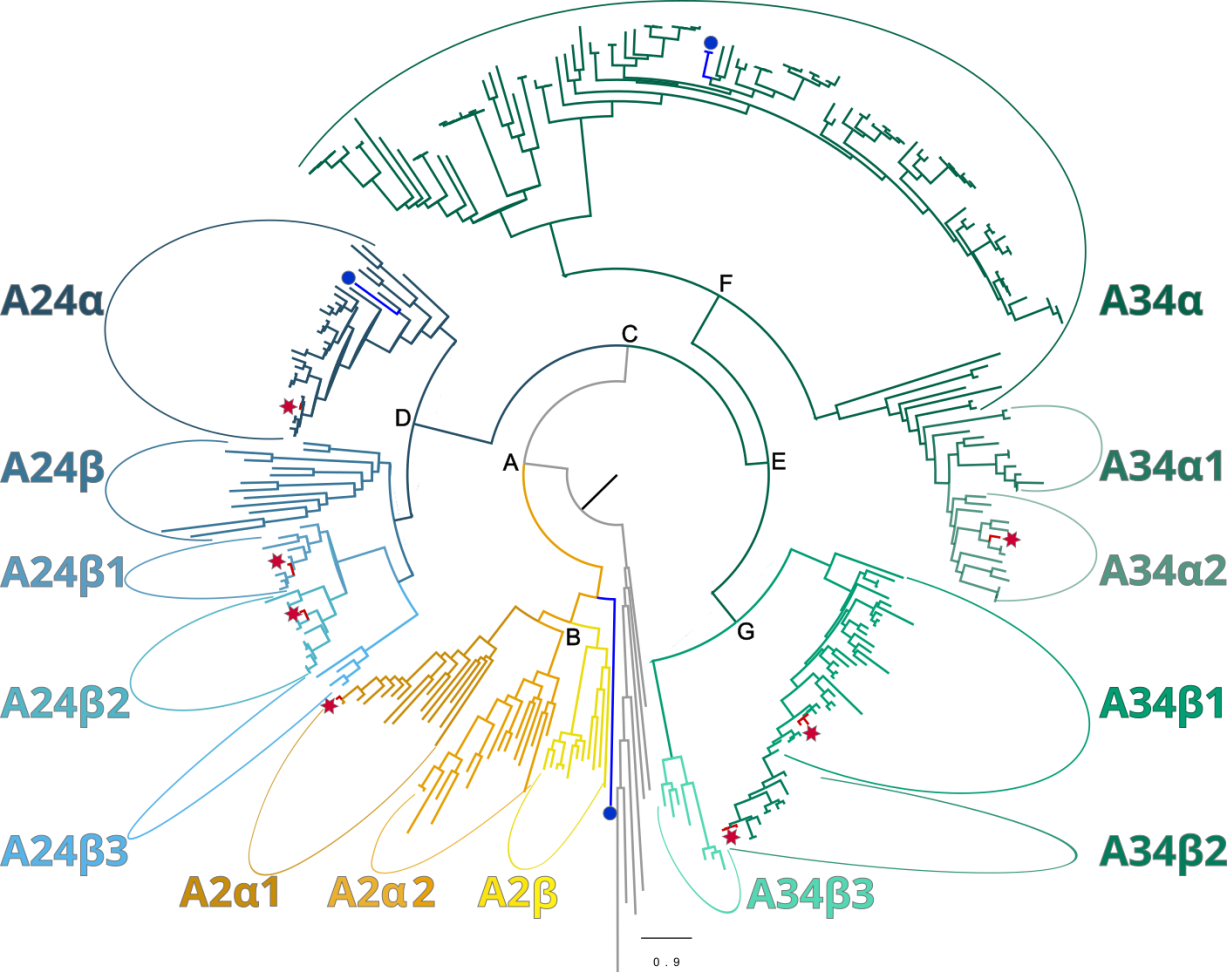
Phylogeny of ITP GPCR homologs in crustaceans, depicted as a circular cladogram, showing the major clades following the *Bombyx mori* nomenclature for class A GPCRs: A2 (yellow), A24 (blue), A34 (green). The position of *Bombyx mori* reference sequences and *Gecarcinus lateralis* homologs are indicated by a blue circle and a red star, respectively. Maximum-likelihood phylogenetic reconstruction was performed with IQtree2 and a JTT+R9 model of evolution, and a total of 424 best Nearest Neighbor Interchange optimization iterations; branch support was assessed via 10,000 UltraFast bootstrap approximations and an aBayes parametric test (see Materials and Methods). Support values for the depicted splits are the following (aBayes/UFboot): A = 1/100; B = 0.999/90; C = 1/100; D = 1/100; E = 0.997/79; F = 1/100; and G = 1/98. Support values within clades are shown in **Figures 2**–**4**. Full annotated phylogeny available in **Supplementary Data 4**.

**Figure 2:**
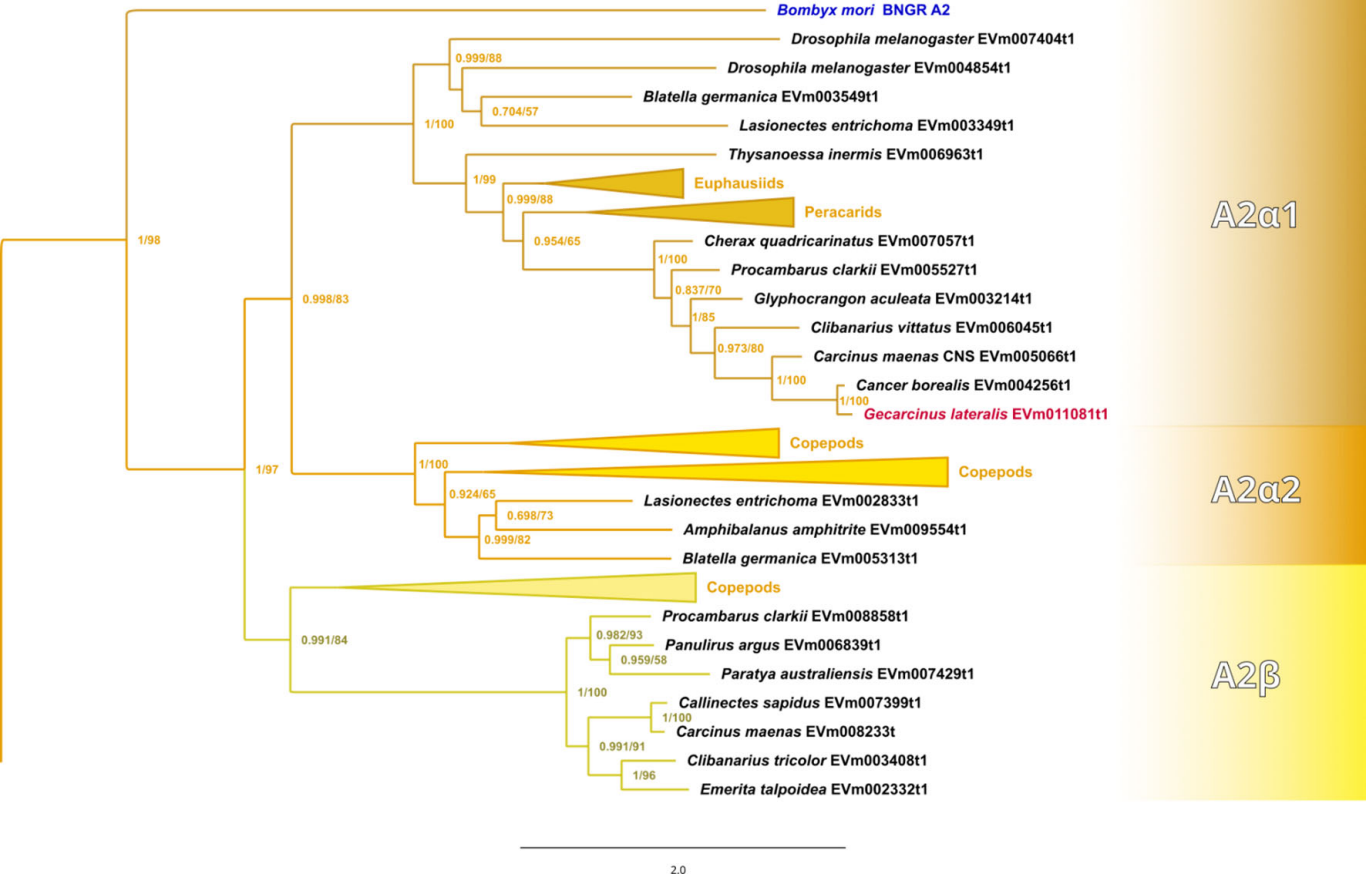
Expanded phylogram of the A2 clade from the phylogenetic tree in **Figure 1**. The *Bombyx mori* Bommo_BNGR_A2 reference sequence and *G. lateralis* homolog are indicated by blue and red font colors, respectively. Maximum-likelihood phylogenetic reconstruction was performed with IQtree2 and a JTT+R9 model of evolution, and a total of 424 best Nearest Neighbor Interchange optimization iterations; branch support was assessed via 10,000 UltraFast bootstrap approximations and an aBayes parametric test. Full annotated phylogeny available in **Supplementary Data 4**.

**Figure 3:**
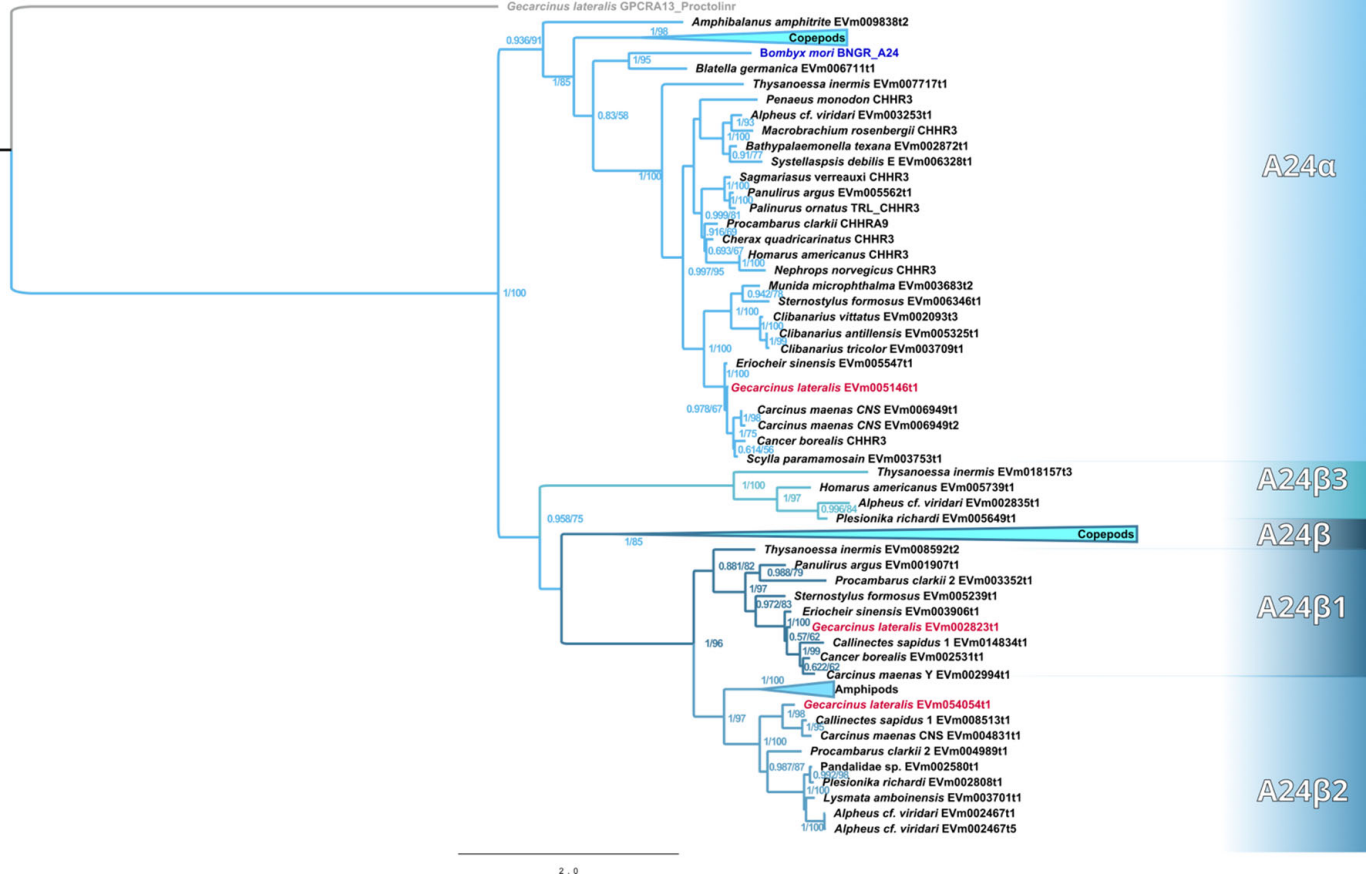
Phylogram of the A24 clade and subclades. The *Bombyx mori* Bommo_BNGR_A24 reference sequence and *G. lateralis* homologs are indicated by blue and red font colors, respectively. Subclades with the β designation represent crustacean-specific lineages that do not include hexapods. Maximum-likelihood phylogenetic reconstruction was performed with IQtree2 and JTT+I+G4 as the best-fit model and a total of 274 best Nearest Neighbor Interchange optimization iterations. Branch support was assessed via 10,000 UltraFast bootstrap approximations and an aBayes parametric test. Full annotated phylogeny available in **Supplementary Data 4**.

**Figure 4:**
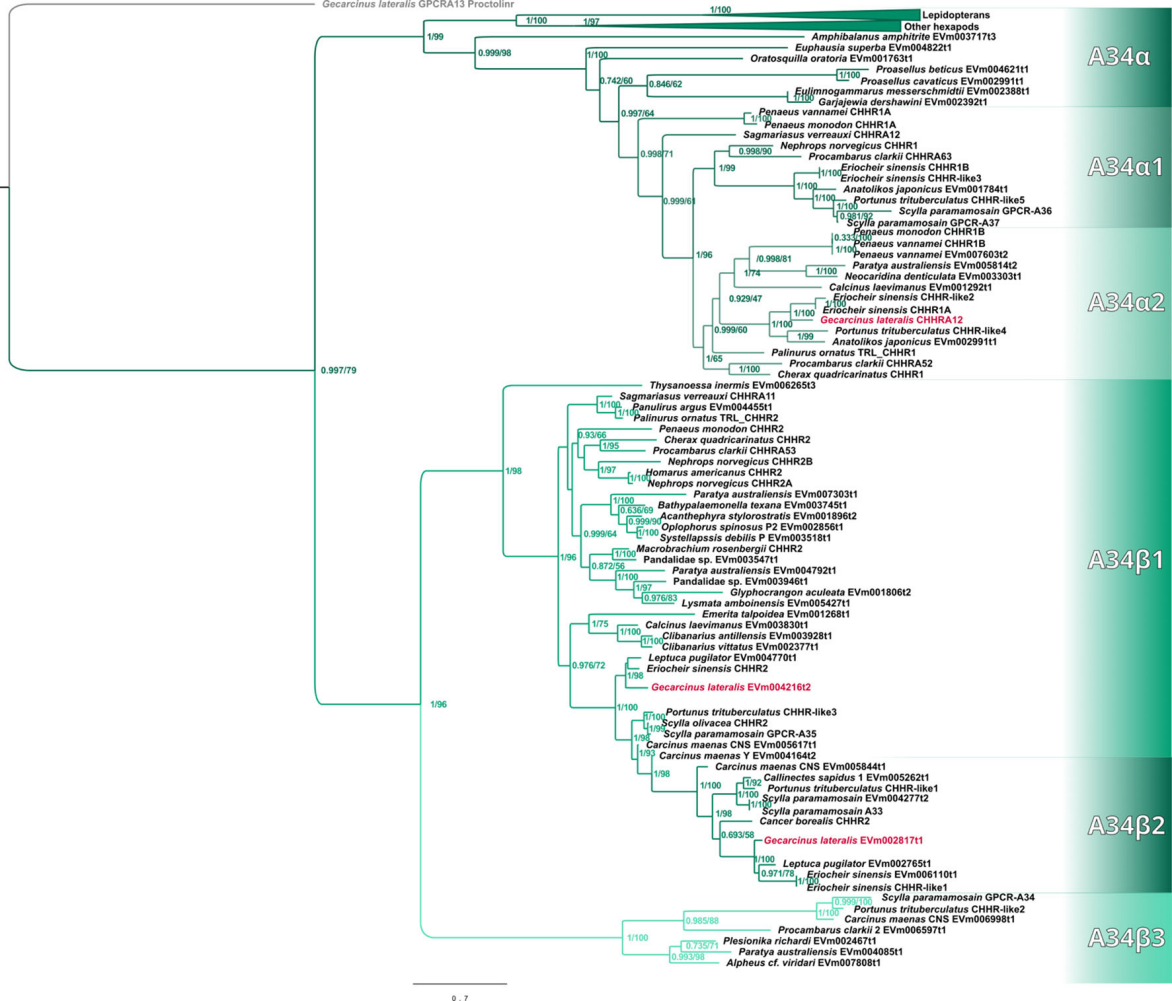
Phylogram of the A34 clade and subclades. The *G. lateralis* homologs are identified by red font color. The *Bombyx mori* Bommo_BNGR_A34 reference sequence is within the collapsed Hexapoda clade. Subclades with the β designation represent crustacean-specific lineages that do not include hexapods. Additionally, subclade A34β2 is restricted to true crabs (*Malacostraca*: *Decapoda*: *Brachyura*). Maximum-likelihood phylogenetic reconstruction was performed with IQtree2 and JTT+F+R6 as the best-fit model, and a total of 685 best Nearest Neighbor Interchange optimization iterations. Branch support was assessed via 10,000 UltraFast bootstrap approximations and an aBayes parametric test. Full annotated phylogeny available in **Supplementary Data 4**.

Maximum-likelihood phylogenetic analyses of the insect ITP and crustacean CFRCs recovered a well-supported phylogeny with three major CFRC clades, which corresponded to *B. mori* BNGR-A2, BNGR-A24, and BNGR-A34 (**Figure 1**) and were represented across Pancrustacea. Each of these major clades in turn contained 14 well-supported subclades that had an uneven taxonomic distribution, suggesting possible specialization events in specific groups. Ten of the 14 subclades had crustacean-only lineages (A2β, A24β, A24β1, A24β2, A24β3, A34α1, A34α2, A34β1, A34β2, & A34β3; **Figure 1**). Clade CFRC-A2 was subdivided into two α subclades and one β subclade (**Figure 1**). CFRC-A2α1 was a large subclade that contained 16 sequences from hexapods and malacostracan crustaceans; CFRC-A2α2 contained 16 sequences from hexapods and copepods; and CFRC-A2β contained 11 sequences from copepods and decapods, but not from hexapods (**Figure 2**). Clade CFRC-A24 was subdivided into one general pancrustacean subclade that included insects and crustaceans (A24α; 31 sequences), a copepod-specific subclade (A24β; 15 sequences), and three malacostracan subclades (A24β1, 9 sequences; A24β2, 10 sequences; and A24β3, 4 sequences; **Figure 3**). The CFRC-A34 clade displayed greater diversity in comparison to the A2 and A24 clades, particularly among lepidopterans and crustaceans, and was subdivided into three α subclades and three β subclades (**Figures 1**, **4**). Subclade CFRC-A34α contained 108 sequences from hexapods and non-decapod malacostracan crustaceans, while subclades A34α1 and A34α2 contained 11 and 14 sequences, respectively, from decapod species only (**Figure 4**). The three A34β subclades represented a remarkable diversification and expansion of CFRCs in decapods (**Figure 4**). A34β1 contained 35 sequences from every decapod infraorder, whereas subclade A34β2 contained 12 sequences from only brachyuran species (**Figure 4**). The relatively smaller A34β3 subclade contained seven sequences from brachyuran, astacidean, and caridean species (**Figure 4**).

Published CFRC sequences, as well as those of additional CFRC sequences, in seven decapod species are summarized in **Table 1**. The sequences are organized according to the proposed classification nomenclature. Most of the published sequences were in the A34 clade; the lone exception was *Pc-GCRC-A9*, which was in the A24 clade (**Table 1**). New contigs encoding A2 and A24 sequences were identified in five species from transcriptomes in the CrusTome database. No new CRFRCs were identified in *P. trituberculatus*, as the RNAseq data for this species was not included in CrusTome (41). CrusTome also did not include transcriptomic data for *S. verreauxi*; the new sequence, designated *Sv-CHHR3*, was provided by Dr. Tomer Ventura for the phylogenetic analysis (**Figure 3**). Unpublished CFRC sequences in the A2 clade were identified in *P. clarkii* (A2α1 and A2β), *C. maenas* (A2α1 and A2β), and *G. lateralis* (A2α1). New A24 CFRC sequences were identified in *P. clarkii* (A24β1 and A24β2), *S. paramamosain* (A24α), *E. sinensis* (A24α and A24β1), *S. verreauxi* (A24α), *C. maenas* (A24α, A24β1, and A24β2), and *G. lateralis* (A24α, A24β1, and A24β2). One new A34 CFRC sequence was identified in *C. maenas* (A34β3).

**Table 1:** Classification of CHH Family GPCR candidates in seven decapod species.

Clade (*Bombyx*)	Previous Classifications and New Sequences Identified in Seven Decapod Species							Proposed Classification
	*P. clarkii* ^1^	*S. paramamosain* ^2^	*P.trituberculatus* ^3^	*E. sinensis* ^4^	*S. verreauxi* ^5^	*C. maenas* ^6^	*G. lateralis* ^7^	
Bommo_BNGR_A2	*Pc-EVm005527t1*					*Cm CNS EVm005066t1*	*Gl-EVm011081t1*	CFRC-A2α1
	*Pc-EVm008858t1*					*Cm CNS EVm008233t1*		CFRC-A2β
Bommo_BNGR_A24	Pc-GPCR-A9	*Sp-EVm003753t1*		*Es-EVm005547t1*	Sv-CHHR3	*Cm CNS EVm003753t1* & *t2*	*Gl-EVm005146t1*	CFRC-A24α
	*Pc-EVm003352t1*			*Es-EVm003906t1*		*Cm Y EVm002994t1*	*Gl-EVm002823t1*	CFRC-A24β1
	*Pc-EVm004989t1*					*Cm CNS EVm008513t1*	*Gl-EVm054054t1*	CFRC-A24β2
Bommo_BNGR_A34	Pc-GPCR-A63	Sp-GPCR-A36/A37	Pt-CHHR-like5	Es-CHHR-like3	Sv-GPCR-A12			CFRC-A34α1
	Pc-GPCR-A52		Pt-CHHR-like4	Es-CHHR-like2			Gl-GPCR-A12	CFRC-A34α2
	Pc-GPCR-A53	Sp-GPCR-A35	Pt-CHHR-like3		Sv-GPCR-A11	569.40694_TR1315|c9_g1_i7	Gl-GPCR-A9	CFRC-A34β1
		Sp-GPCR-A33	Pt-CHHR-like1	Es-CHHR-like1		13948.1_TR1315|c9_g1_i1	Gl-GPCR-A10	CFRC-A34β2
		Sp-GPCR-A34	Pt-CHHR-like2			*Cm CNS EVm006998t1*		CFRC-A34β3
								

Previous names of receptor sequences are compared with the proposed CFRC nomenclature. New sequences obtained from the CrusTome database are italicized. Sequences are provided in **Supplementary Data 2**. BNGR, *Bombyx* neuropeptide G protein-coupled receptor; CFRC, CHH family receptor candidate; CHHR, CHH receptor; and GPCR, G protein-coupled receptor.

^1^ *Procambarus clarkii*. From (19).

^2^ *Scylla paramamosain*. From (22). Sp-GPCR-A36 and -A37 appear to be isoforms generated by alternative splicing.

^3^ *Portunus trituberculatus*. From (22).

^4^ *Eriocheir sinensis*. From (22).

^5^ *Sagmariasus verreauxi*. Sv-GPCR-A11 and -12 from (21). Sv-CHHR3 sequence provided by T. Ventura.

^6^ *Carcinus maenas. YO transcriptome*. From (23).

^7^ *Gecarcinus lateralis*. From (17). In reference (6), Gl-GPCR-A9 was designated as Gl-CHHR1A; Gl-GPCR-A10 was designated as Gl-CHHR1B; and Gl-GPCR-A12 was designated as Gl-CCHR2.

Data for the *G. lateralis* and *C. maenas CFRC* contig sequences are summarized in **Tables 2**, **3**, respectively. Most of the new *CFRC* contigs were expressed in nervous tissues. The four new *Gl-CFRC* contigs (*Gl-CFRC-A2α1*, *-A24α*, *-A24β1*, and *-A24β2*) were obtained from the eyestalk ganglia (ESG) transcriptome; none were obtained from the YO transcriptome (**Table 2**). Five of the six new *Cm-CFRC* contigs (*Cm-CFRC-A2α1*, *-A2β*, *-A24α*, *-A24β2*, and *-A34β3*) were obtained from the central nervous system (CNS) transcriptome; *Cm-A24β1* was obtained from the YO transcriptome (**Table 3**). A full-length sequence of *Gl-CFRC-A34α2* (formerly *Gl-CHHR-A12*) was not extracted from the CrusTome database; two overlapping partial sequences from the *G. lateralis* YO transcriptome were used to construct the complete protein (**Table 2**) (17). The previously-identified *Gl-CFRC-A34β1*, *Cm-CFRC-A34β1*, and *Gl-CFRC-A34β2* sequences were present in both nervous tissue and YO, whereas the *Cm-A34β2* sequence was only present in the CNS (**Tables 2**, **3**).

**Table 2:** Properties of *G. lateralis* contigs encoding CHH family receptor candidates (CFRCs).

Gene	Contig Number(s)	Contig Length (bp)^1^	ORF (aa)^1^	GenBank Accession #
*Gl-CFRC-A2α1*	ESG: GeclatEVm011081t1	872	272*	OR671212
*Gl-CFRC-A24α*	ESG: GeclatEVm005146t1	2533	476	OR671213
*Gl-CFRC-A24β1*	ESG: GeclatEVm002823t1	2623	687	OR671214
*Gl-CFRC-A24β2*	ESG: GeclatEVm054054t1	691	134*	OR671215
*Gl-CFRC-A34α2*	CHHR-A12^2^	1242	499	OR671216
*Gl-CFRC-A34β1*	ESG: GeclatEVm004216t2 YO: GeclatEVm004179t1	2657 1927	453 549	OR671217 OR671218
*Gl-CFRC-A34β2*	ESG: GeclatEVm002817t1 YO: GeclatEVm005819t1	3435 3161	688 452*	OR671219 OR671220

DNA and amino acids sequences of the contigs are given in **Supplementary Data 2**. aa, amino acids; bp, base pairs; ESG, eyestalk ganglia; and YO, Y-organ.

^1^Asterisk (*) indicates partial sequence; ORF incomplete.

^2^CHHR-A12 sequence constructed from two overlapping partial contigs in the *G. lateralis* YO transcriptome (17, 43).

**Table 3:** Properties of *C. maenas* contigs encoding CHH family receptor candidates (CFRCs).

Gene	Contig Number(s)	Contig Length (bp)^1^	ORF (aa)^1^	GenBank Accession #
*Cm-CFRC-A2α1*	CNS: CarmaC_EVm005066t1	2581	569	OR671221
*Cm-CFRC-A2β*	CNS: CarmaC_EVm008233t1	2800	425	OR671222
*Cm-CFRC-A24α*	CNS: CarmaC_EVm006949t1 CNS: CarmaC_EVm006949t2	3406 3586	476 380*	OR671223 OR671224
*Cm-CFRC-A24β1*	YO: CarmaY_EVm002994t1	2553	709	OR671225
*Cm-CFRC-A24β2*	CNS: CarmaC_EVm004831t1	2661	585	OR671226
*Cm-CFRC-A34β1*	CNS: CarmaC_EVm005617t1 YO: CarmaY_EVm004164t2	1900 2279	537 596	OR671227 OR671228
*Cm-CFRC-A34β2*	CNS: CarmaC_EVm005844t1	2033	525	OR671229
*Cm-CFRC-A34β3*	CNS: CarmaC_EVm006998t1	2894	474	OR671230

DNA and amino acids sequences of the contigs are given in **Supplementary Data 2**. aa, amino acids; bp, base pairs; CNS, central nervous system; and YO, Y-organ.

^1^Asterisk (*) indicates partial sequence; ORF incomplete.

Contigs encoding CFRC sequences were identified in the transcriptomes from 37 decapod species representing six infraorders. The decapod sequences were assigned to 11 of the 14 subclades (**Table 4**). The three subclades lacking decapod CFRC sequences were A2α2 (**Figure 2**), A24β (**Figure 3**), and A34α (**Figure 4**). None of the 37 decapod species expressed all 11 CFRCs. The number of CFRC sequences for a species ranged from one in *Neocaridina denticulata*, *Munida micropththalma*, and *Acanthephyra stylorostratis* to nine in *P. clarkii*. *CFRC-A24α* and *CFRC-A34β1* were the most common, with *CFRC-A24α* identified in 22 species and *CFRC-A34β1* identified in 27 species from all six infraorders (**Table 4**). Members of the A2 subclades (*A2α1* and *A2β*) were identified in seven species each, but not always in the same species; only in *C. maenas* and *P. clarkii* were both *A2α1* and *A2β* expressed. *A24β3* was the least common sequence in the A24 clade; it was identified in only three species (one astacidean and two carideans). *CFRC-A34α1* was identified in nine species from four infraorders, whereas *CFRC-A34α2* was identified in 12 species from five infraorders (**Table 4**). *CFRC-A34β2* was identified only in the Brachyura, with six of the 10 species expressing both *A34β1* and *A34β2*. *CFRC-A34β3* was the least common of the sequences in the A34 subclades; it was identified in only seven of the 37 species (**Table 4**).

**Table 4:** Classification of CHH family receptor candidates in decapod species.

**Species**	**A2α1**	**A2β**	**A24α**	**A24β1**	**A24β2**	**A24β3**	**A34α1**	**A34α2**	**A34β1**	**A34β2**	**A34β3**
**Brachyura:**											
***A. japonicus***	**0**	**0**	**0**	**0**	**0**	**0**	**X**	**X**	**0**	**0**	**0**
***C. borealis***	**X**	**0**	**X**	**X**	**0**	**0**	**0**	**0**	**0**	**X**	**0**
***C. maenas***	**X**	**X**	**X**	**X**	**X**	**0**	**0**	**0**	**X**	**X**	**X**
***C. sapidus***	**0**	**X**	**0**	**X**	**X**	**0**	**0**	**0**	**0**	**X**	**0**
***E. sinensis***	**0**	**0**	**X**	**X**	**0**	**0**	**X**	**X**	**X**	**X**	**0**
***G. lateralis***	**X**	**0**	**X**	**X**	**X**	**0**	**0**	**X**	**X**	**X**	**0**
***L. pugilator***	**0**	**0**	**0**	**0**	**0**	**0**	**0**	**0**	**X**	**X**	**0**
***P. trituberculatus***	**0**	**0**	**0**	**0**	**0**	**0**	**X**	**X**	**X**	**X**	**X**
***S. olivacea***	**0**	**0**	**0**	**0**	**0**	**0**	**0**	**0**	**X**	**0**	**0**
***S. paramamosain***	**0**	**0**	**X**	**0**	**0**	**0**	**X**	**0**	**X**	**X**	**X**
											
**Astacidea:**											
***C. quadricarinatus***	**X**	**0**	**X**	**0**	**0**	**0**	**0**	**X**	**X**	**0**	**0**
***H. americanus***	**0**	**0**	**X**	**0**	**0**	**X**	**0**	**0**	**X**	**0**	**0**
***N. norvegicus***	**0**	**0**	**X**	**0**	**0**	**0**	**X**	**0**	**X**	**0**	**0**
***P. clarkii***	**X**	**X**	**X**	**X**	**X**	**0**	**X**	**X**	**X**	**0**	**X**
											
**Anomura:**											
***C. antilillensis***	**0**	**0**	**X**	**0**	**0**	**0**	**0**	**0**	**X**	**0**	**0**
***C. tricolor***	**0**	**X**	**X**	**0**	**0**	**0**	**0**	**0**	**0**	**0**	**0**
***C. vittatus***	**X**	**0**	**X**	**0**	**0**	**0**	**0**	**0**	**X**	**0**	**0**
***C. laevimanus***	**0**	**0**	**0**	**0**	**0**	**0**	**0**	**X**	**X**	**0**	**0**
***E. talpoidea***	**0**	**X**	**0**	**0**	**0**	**0**	**0**	**0**	**X**	**0**	**0**
***M. microphthalma***	**0**	**0**	**X**	**0**	**0**	**0**	**0**	**0**	**0**	**0**	**0**
***S. formosus***	**0**	**0**	**X**	**X**	**0**	**0**	**0**	**0**	**0**	**0**	**0**
											
**Penaeoidea:**											
***L. vannamei***	**0**	**0**	**0**	**0**	**0**	**0**	**X**	**X**	**0**	**0**	**0**
***P. monodon***	**0**	**0**	**X**	**0**	**0**	**0**	**X**	**X**	**X**	**0**	**0**
											
**Caridea:**											
***A. cf. viridari***	**0**	**0**	**X**	**0**	**X**	**X**	**0**	**0**	**0**	**0**	**X**
***A. stylorostratis***	**0**	**0**	**0**	**0**	**0**	**0**	**0**	**0**	**X**	**0**	**0**
***B. texana***	**0**	**0**	**X**	**0**	**0**	**0**	**0**	**0**	**X**	**0**	**0**
***G. aculeata***	**X**	**0**	**0**	**0**	**0**	**0**	**0**	**0**	**X**	**0**	**0**
***L. amboinensis***	**0**	**0**	**0**	**0**	**X**	**0**	**0**	**0**	**X**	**0**	**0**
***M. rosenbergii***	**0**	**0**	**X**	**0**	**0**	**0**	**0**	**0**	**X**	**0**	**0**
***N. denticulata***	**0**	**0**	**0**	**0**	**0**	**0**	**0**	**X**	**0**	**0**	**0**
***O. spinosus***	**0**	**0**	**0**	**0**	**0**	**0**	**0**	**0**	**X**	**0**	**0**
***P. australiensis***	**0**	**X**	**0**	**0**	**0**	**0**	**0**	**X**	**X**	**0**	**X**
***P. richardi***	**0**	**0**	**0**	**0**	**X**	**X**	**0**	**0**	**0**	**0**	**X**
***S. debilis***	**0**	**0**	**X**	**0**	**0**	**0**	**0**	**0**	**X**	**0**	**0**
											
**Palinuroidea:**											
***P. argus***	**0**	**X**	**X**	**X**	**0**	**0**	**0**	**0**	**X**	**0**	**0**
***P. ornatus***	**0**	**0**	**X**	**0**	**0**	**0**	**0**	**X**	**X**	**0**	**0**
***S. verreauxi***	**0**	**0**	**X**	**0**	**0**	**0**	**X**	**0**	**X**	**0**	**0**

“X” indicates presence and “0” indicates not identified in the CrusTome database. See **Supplementary Data 2**.

The colors in **Table 4** match the colors in the trees for the A2, A24, and A34 clades (**Figures 1**–**4**).

### Sequence analysis of the ECL2, ICL3, and ECL3 regions of decapod CFRCs

**Figure 5:**
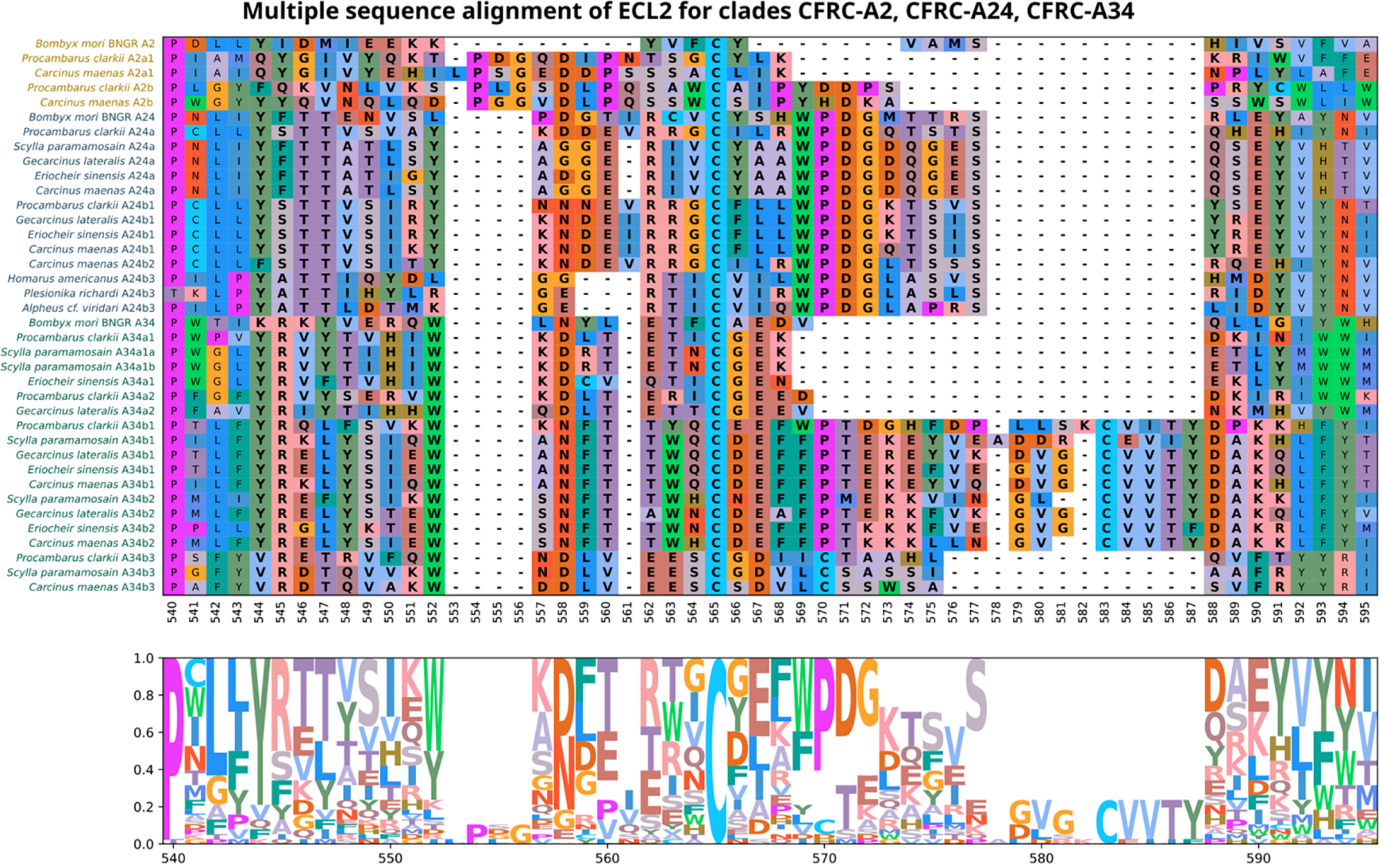
Multiple sequence alignments of the Extracellular Loop 2 (ECL2) sequences in clades A2, A24, and A34 of *Bombyx mori* ITP GPCR homologs, which depicts the diversity of ECL2 types found in decapod crustaceans. ECL2 residues are highlighted in bold. MSA color scheme corresponds to similarities in physicochemical properties of amino acid residues (see Materials and Methods). All the CFRC sequences have a highly conserved cysteine at reference position #565.

Multiple sequence alignment of the ECL2 region compared the sequence content, conservation, and the annotation of putative novel structures in decapod CFRCs with the *B. mori* BNGR-A2, -A24, and -34 sequences. A common feature shared by all the CFRCs, including *Bombyx*, was a conserved cysteine (C) in ECL2 (**Figure 5**, reference alignment position #565). The A2, A24, and A34 CFRC clades displayed unique ECL2 amino acid compositions that were consistent across taxa and within each subclade as depicted by the alignments and logo plots (**Figure 5**, reference alignment positions #544 to #591). The A2α1 and A2β sequences had an insertion of three or four amino acids unique to the A2 clade (**Figure 5**, reference alignment positions #553 to #556). All the A24 sequences, including BNGR-A24, had a pair of threonine residues (T) at reference positions #546 and #547, and a nine amino acid sequence (WPDGxxxxS), starting four residues C-terminal to the conserved cysteine (**Figure 5**, reference alignment positions #569 to #577). The A24α, A24β1, and A24β2 subclades had a conserved tyrosine (Y) that distinguished them from the A24β3 sequences (**Figure 5**, reference alignment position #552). All the A34 sequences, including BNGR-34, had a conserved tryptophan (W) eight residues N-terminal to the conserved cysteine (**Figure 5**, reference alignment position #552). Within the A34 clade, the A34α subclade had the shortest ECL2 sequence, while the A34β1/β2 subclades had the longest ECL2 sequence (**Figure 5**). The length of the A34β3 ECL2 sequence was intermediate between A34α and A34β1/β2. Moreover, the ECL2 sequences of the A34β subclades had a second conserved cysteine (C) absent in the A34α ECL2 sequences (**Figure 5**; cysteine located at reference alignment position #583 in A34β1/β2 and at reference alignment position #570 in A34β3).

**Figure 6:**
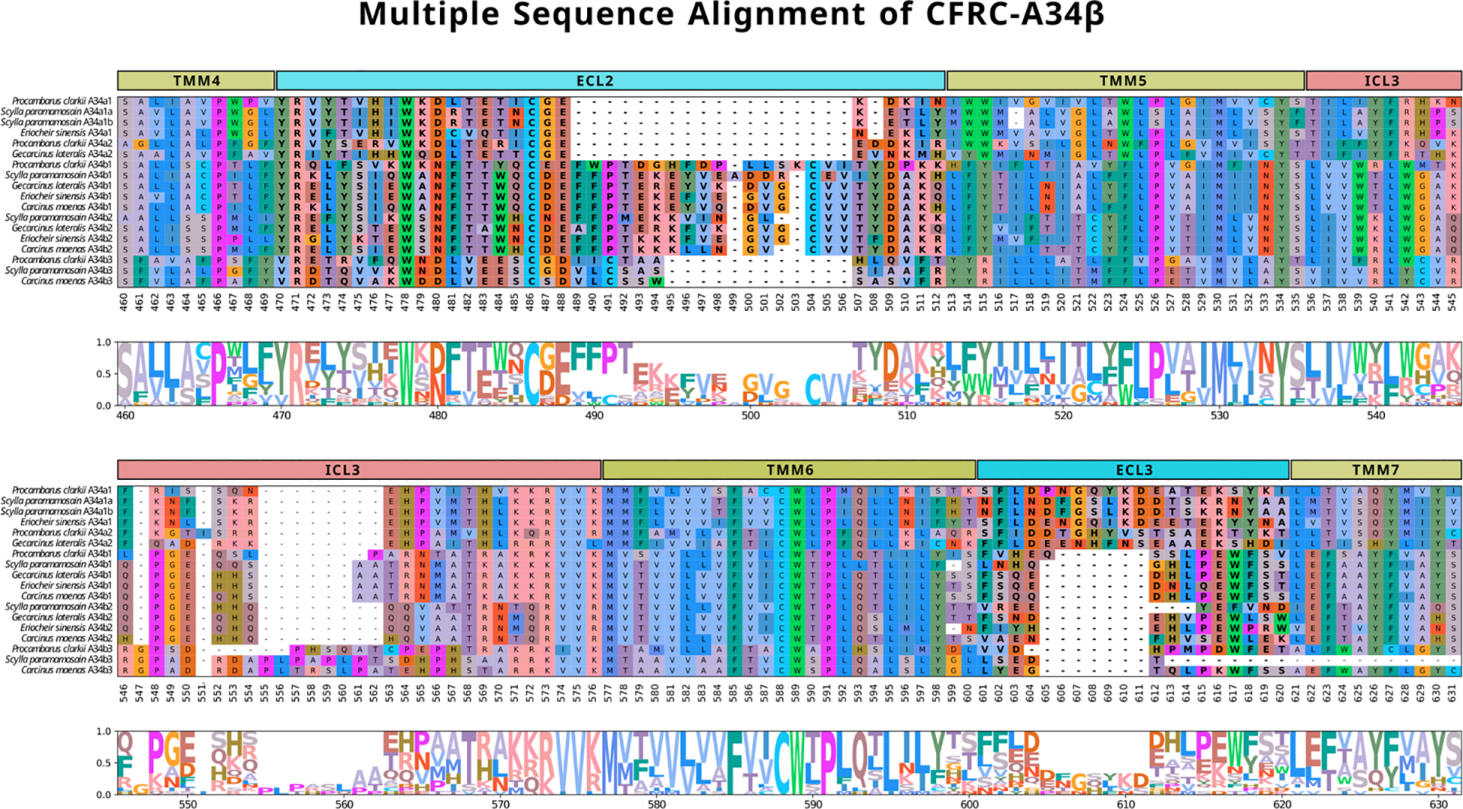
Multiple sequence alignments of the ECL2, TMM5, ICL3, TMM6, and ECL3 regions across subclades A34β1, A34β2, and A34β3 in representative decapod species (*Procambarus clarkii*, *Scylla paramamosain*, *Eriocheir sinensis*, *Gecarcinus lateralis*, and *Carcinus maenas*). The alignment illustrates the composition and length of the ECL regions that reflect putative differences in ligands and/or binding affinities. Note that the sequence reference numbers differ from those in **Figure 5** due to the sequences selected for the alignments. ECLs are highlighted in bold. MSA color scheme corresponds to similarities in physicochemical properties of amino acid residues (see Materials and Methods).

Brachyuran A34 sequences were selected for multiple sequence alignment to compare the ECL2, ECL3, ICL3, TMM5, and TMM6 regions in greater detail. Crayfish (*P. clarkii*) sequences (Pc-CFRC-A34α1, -A34α2, -A34β1, and -A34β3) were included for reference. All the A34 ECL2 sequences had a conserved arginine (R), tryptophan (W), and cysteine (C) located at reference alignment positions #471, #478, and #486, respectively, as shown on the CFRC-A34 clade-specific alignment (**Figure 6**). All the CFRCs had a conserved CWxP motif in TMM6 (**Figure 6**; reference alignment positions #588 to #591). The five A34 subclades (A34α1, A34α2, A34β1, A34β2, and A34β3) were differentiated by amino acid sequence and length of the ICL3 region (**Figure 6**). The A34β3 subclade was distinguished from the other A34 subclades by a seven amino acid sequence between the tryptophan (W) and the cysteine (C) in ECL2 (WxDLVEESC; **Figure 6**, reference alignment positions #478 to #486) and by a four or six amino acid insertion in ICL3 (**Figure 6**; reference alignment positions #555 to #560). The A34β1/β2 subclades had a 14- to 16-amino acid insertion containing a second conserved cysteine (C) (**Figure 6**, reference alignment positions #489 to #506). This insertion forms a second two-stranded β-sheet (see “Structural modeling of Gl-CFRC-A24 and -A34 proteins” section below). Additionally, the ECL2 region of the brachyuran A34β1/β2 subclades had a conserved 14-amino acid sequence that included the first cysteine (WxNFTxWxCxExFP; **Figure 6**, reference alignment positions #478 to #491; **Supplementary Data 4**). The A34α1/α2 sequences had a six-amino acid insertion in ECL3 that was not present in the A34β subclades (**Figure 6**, reference alignment positions #606 to #611).

**Table 5** summarizes the conserved motifs identified via multiple sequence alignments in ECL2 and ECL3 that distinguish the 11 decapod CFRC subclades. The analysis included the CFRC sequences from 37 decapod species, with the number of sequences analyzed ranging from three for A24β3 to 27 for A34β1 (**Table 4**). All the ECL2 motifs contained the conserved cysteine (**Table 5**). CFRC-A2α1 and -A2β had four conserved amino acid residues (PxGxDxP; **Table 5**), which distinguished them from the members of the A24 and A34 clades. The four A24 subclades (CFRC-A24α, -A24β1, -A24β2, and -A24β3) had “WPDG” in the ECL2 motif that was absent in the A2 and A34 subclade sequences (**Table 5**). These ECL2 and ECL3 motifs distinguished the CFRC-A24α subclade from the CFRC-A24β subclades. The CFRC-A24β1 and -A24β2 sequences showed similarities, with “STTVS” in ECL2 and “HNS” and “IQH” in ECL3 (**Table 5**). CFRC-A24β3 ECL3 motif was similar to CFRC-A24β1 and -A24β2 but differed from CFRC-A24β1/β2 in the ECL2 motif in both length and amino acid composition. The ECL2 and ECL3 motifs among the three CFRC-A34 subclades varied in length and sequence. CFRC-A34β1 and -A34β2 had a longer ECL2 with a second conserved cysteine (**Table 5**). The “CVVTxDAK” sequence of this region in CFRC-A34β2 only occurred in brachyurans (**Table 5**). CFRC-A34β3 showed an ECL2 motif length closer to that of CFRC-A34α than those of CFRC-A34β1/β1. Similar to CFRC-A34β1/β2, the CFRC-A34β3 ECL2 motif sequence had a second conserved cysteine (**Table 5**).

**Table 5:** Sequences of conserved motifs in the ECL2 and ECL3 of decapod CFRCs.

CFRC Clade	ECL2 Motif Sequences	ECL3 Motif Sequences
***CFRC-A2α1***	QYGIVYxxx[L-]PxGxDxPxxSx **C** xxKxxxx	HxDxxNPxxVxLFNx
***CFRC-A2β***	xxxVNxxxxPxGxDLPQSxW **C** xIPYxDxxxxxx	xxxTxxxQWTxxxxSVNTx
***CFRC-A24α***	YxTTxxxxYxxxE[x-]Rxx **C** xxxWPDGxxxxSQxEx	xxxxIxxxxxIQx
***CFRC-A24β1***	YSTTVSIxYxNxEIRRG **C** FLLWPDGxTSxSYxEY	HNSQVLDxxxIQH
***CFRC-A24β2***	xSTTVSxxYKxDEVRRG **C** ILRWPDGxTSxSxxEH	HNSxxLxTAHIQH
***CFRC-A24β3***	YATTxxxxxGxRTI **C** VIxWPDGLAxxSxxDY	HHPQLSxRPYVQH
***CFRC-A34α1***	YRVxTxHIWKDxxxTx **C** GExxxxx	xFLxxxGxxKDxxxxxxYxx
***CFRC-A34α2***	YRxYxxxxWxDLTExx **C** GEExxKxx	xFxDxxxHxxSxxAEKxxxx
***CFRC-A34β1***	YRxLxSxxWxNFTTxQ **C** xEFxPTxxxxxxxxxx[x-]CxxxYDxKx	F[x-]xxxxxLPEWFSx
***CFRC-A34β2***	YRxxYxxxWSNFTxWx **C** xExFPxxxxxxxGx[G-]CVVTxDAKx	xxxx[x-]HxxEWxxx
***CFRC-A34β3***	VRxTxVxxWxDLVEES **C** xDxxCxxxxxxxFx	x[ED]xxxxPxWFxx

The ECL2 motifs are centered around a conserved Cys (**C**). A second cysteine in CFRC-A34β1, -A34β2, and -A34β3 is indicated with double underline. Sequences shared between two or more of the A2 or A24 CFRCs are indicated with double underline. Brackets indicate sites with possible indels or residues seen in equal proportions.

### Multiple sequence alignments and structural modeling of Gl-MIH and Gl-CHH proteins

Conserved amino acids in the *G. lateralis* MIH and eyestalk CHH sequences (70, 71) were identified by multiple sequence alignments with MIH mature peptides from 33 brachyuran species and eyestalk CHH isoform mature peptides from 48 brachyuran species (data not shown). The neuropeptides had six conserved cysteines located at positions #7, #24, #27, #40, #44, and #53 in MIH and at positions #7, #23, #26, #39, #43, and #52 in CHH, which is characteristic of the CHH superfamily (**Table 6**) (8, 9). MIH had a conserved Gly12 that was absent in CHH. Nine hydrophobic residues, which stabilize peptide structure (12), are indicated in **Table 6**. MIH and CHH differed in the number and lengths of the α-helical regions. CHH had four α-helices, while MIH had five α-helices, with the additional short α1/3_10_-helical turn before the Gly12 (**Table 6**).

**Table 6:** Consensus sequences of brachyuran MIH and eyestalk CHH isoform mature peptides showing conserved amino acids.

MIH	
Gl-MIH: _a1__ _________a2____________ ___a3__ ______a4___ ________a5_______ A**VI**N**D**ECPNV**I**GN**R**D**I**F**K**K**V**D**WI** CEDCANIF**R**IDGLATLCRKNC **F**RNID**F**L**W** C **V**YAS**E**RQAEKDE**L**TRY**V**SI**L**R**AG**SV 1 10 20 30 40 50 60 70 78
Consensus sequence: xVxNDxCPNxIGNRDxxKxVxWICxDCxNIxRxxGxAxxCRxxCFxNxDFxWCVxAxERxxxxxxLxxxVxILxAGxx 1 10 20 30 40 50 60 70 78
CHH
Gl-CHH: ___________a2__________ ____a3___ _______a4___ ______a5_________ **Q**IYDRSC **K**GV**Y**D**R**S**L**FNK**L**EH**V** CDDCYNL**YR**TSFVYSSCRENC **Y**SNLV**F**RQC **M**EDLLLMDVFDE**Y**AKA**V**QV**V**GRKKK 1 10 20 30 40 50 60 70 77
Consensus sequence: QxxDxxCKGxYDRxxFxxLxxVCxDCYNLYRxxxVxxxCRxNCYxNxVxRQCxxDLLxxDxxxxxxxxxQxVGxKxx 1 10 20 30 40 50 60 70 77

**Figure 7:**
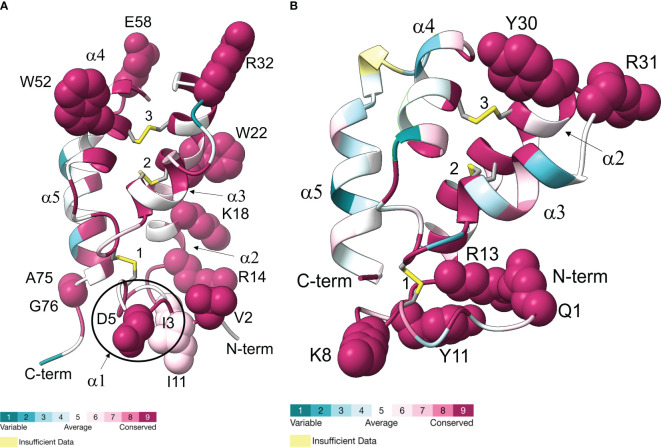
Ribbon diagrams representing Gl-MIH **(A)** and Gl-CHH **(B)** are shown with colors ranging from blue to magenta based on residue conservation. Three conserved disulphide bridges are shown in stick representation; numbers identify the cysteines (Gl-MIH: #1, C7-C44; #2, C24-C40; and #3, C27-C53) (Gl-CHH: #1, C7-C43; #2, C23-C39; and #3, C26-C52). The α-helices are labelled (α1, α2, α3, α4, and α5). The surfaced-exposed conserved residues are shown as spheres (see **Table 6**). Gl-MIH had an additional short α1-helix, specifically 3_10_-helix, at the N-terminus (circled) that is absent from Gl-CHH. The C-terminal four amino acids of Gl-CHH (RKKK) were not included in the modeling. Molecular graphics images were produced using the Chimera package (see Materials and Methods). Hydrogens are not shown for clarity. Coordinates of the AlphaFold2 models in PDB format are available in **Supplementary Data 6**.

Gl-MIH and Gl-CHH sequences (70, 71) and locations of α-helical regions (a1, a2, a3, a4, and a5) are indicated by lines above the sequences. The six conserved cysteines are underlined. Surface-exposed conserved amino acids in the Gl-MIH and Gl-CHH sequences are indicated by red bold font. Nine conserved hydrophobic residues that stabilize peptide conformation are indicated by blue bold font. The four amino acids (RKKK) at the C-terminus of Gl-CHH were not included in the modeling (**Figure 7**).

Structural models of Gl-MIH and the eyestalk Gl-CHH isoform mature peptides are shown in **Figure 7**. Conserved surface-exposed residues from **Table 6** were included in the Gl-MIH and Gl-CHH structural models, as these residues have roles in their structure, function, and protein-receptor interactions (9, 72). The N-terminal sequences of Gl-MIH and Gl-CHH were conserved across other brachyuran species and were usually found on the external surface of the protein (**Table 6**; **Figure 7**). The C-terminal region of Gl-CHH, which included the α4- and α5-helices, was less conserved in comparison to its N-terminal region and the C-terminal region of Gl-MIH (**Figure 7**). Gl-MIH also had a longer and slightly disordered C-terminus, compared to Gl-CHH, and had two conserved surface exposed residues (Ala75 and Gly76). Three conserved disulphide bridges were present in Gl-MIH (Cys7-Cys44, Cys24-Cys40, and Cys27-Cys53) and Gl-CHH (Cys7-Cys43, Cys23-Cys39, and Cys26-Cys52). As noted above, Gl-MIH had an additional short α1/3_10_-helix at Pro8 to Ile11 that was absent in Gl-CHH (**Figure 7A**). Consistent with the literature and the solution structure of MIH from the Kuruma prawn (*Marsupenaeus japonicus*), the C-terminal region of MIH was located close to α1/3_10_-helix in the tertiary structure (**Figure 7A**) (12). The α2-helix of Gl-MIH contained three basic (Arg14, Lys18, and Arg32) residues and an aromatic hydrophobic (Trp22) residue that were surface-exposed and conserved (**Figure 7A**). The disordered N-terminal region of Gl-CHH consisted of surface-exposed and conserved residues Gln1, Lys8, Tyr11, and Arg13 (**Figure 7B**). The C-terminal α5-helices of Gl-MIH and GI-CHH lacked surface-exposed conserved residues. However, GI-MIH had two surface-exposed conserved residues (Ala75 and Gly76) in the flexible loop following the α5-helix (**Figure 7A**). The region between the α3- and α4-helices had surface-exposed residues in Gl-MIH (Arg32, Trp52 and Glu58) and in Gl-CHH (Tyr30 and Arg31) (**Figure 7**).

### Structural modeling of Gl-CFRC-A24 and -A34 proteins

**Figure 8:**
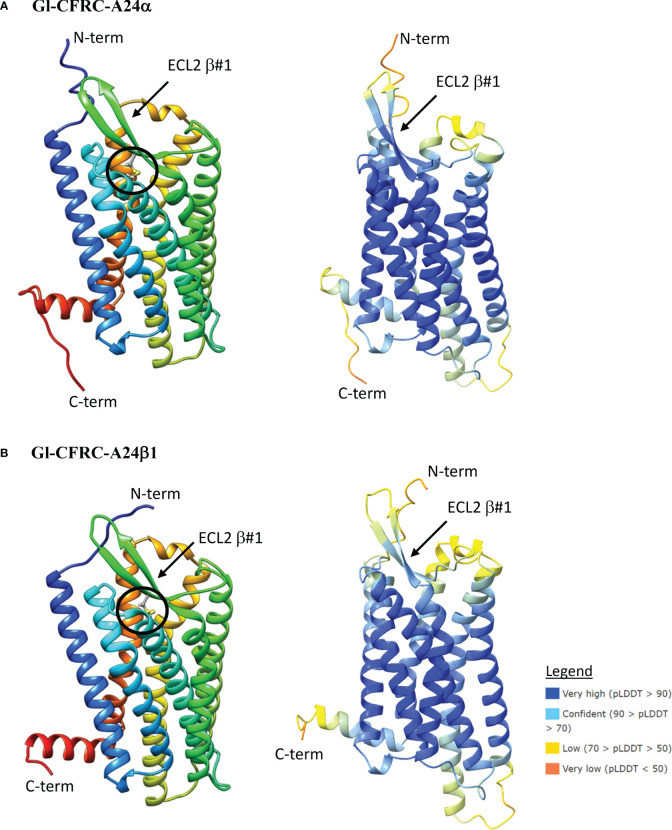
Structural models of the *G. lateralis* A24α **(A)** and A24β1 **(B)** receptors. On the left, ribbon diagrams are shown with colors ranging from blue for the N-terminus to red for the C-terminus. Images were produced using the Chimera package. On the right, three-dimensional prediction using AlphaFold2 (see Materials and Methods). Per-residue confidence score (pLDDT) designates the estimation of confidence on a scale from 0 to 100, with colors representing pLDDT confidence scores from very low (orange) to very high (dark blue; see legend). All receptors showed a common topology of seven transmembrane (TMM) α-helices connected by three extracellular loops (ECLs) and three intracellular loops (ICLs). The N-terminus is in the extracellular space and the C-terminus is in the cytosol. The ECL2 has a two-stranded β-sheet, designated β#1. The disulfide bridge that anchors the ECL2 β#1 to TMM6 is shown as ball and sticks (circle), which are located between C148 and C227 in Gl-CFRC-A24α and between C178 and C258 in Gl-CFRC-A24β1.

**Figure 9:**
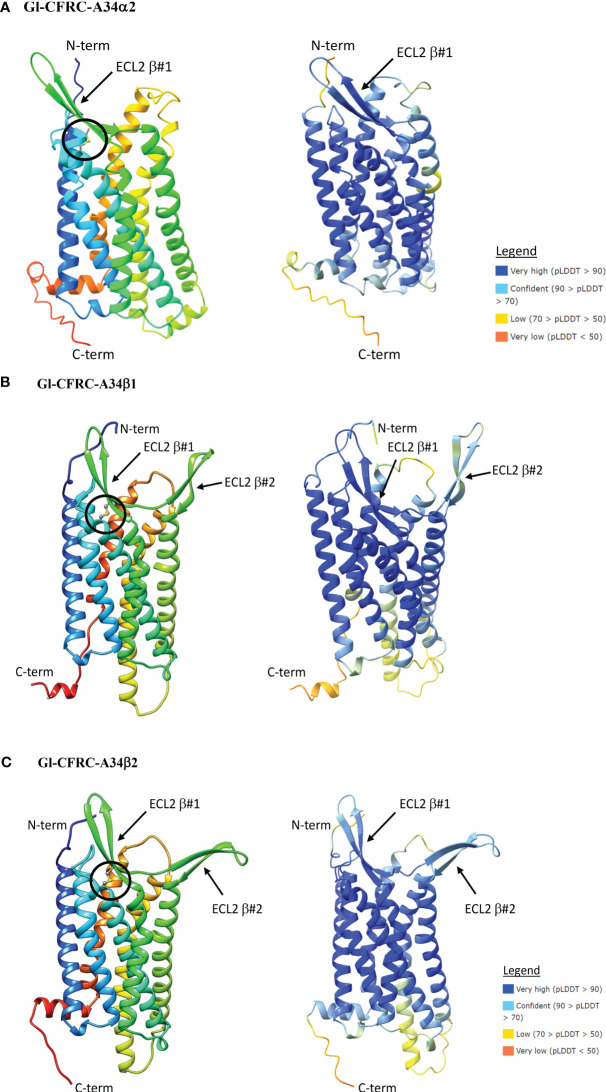
Structural models of the *G. lateralis* A34α **(A)**, A34β1 **(B)**, and A34β2 **(C)** receptors. On the left, ribbon diagrams are shown with colors ranging from blue for the N-terminus to red for the C-terminus. Images were produced using the Chimera package. On the right, three-dimensional prediction using AlphaFold2. Per-residue confidence score (pLDDT) designates the estimation of confidence on a scale from 0 to 100, with colors representing pLDDT confidence scores from very low (orange) to very high (dark blue; see legend). All receptors showed a common topology of seven transmembrane α-helices connected by three ECLs and three ICLs with the N-terminus is in the extracellular space and the C-terminus is in the cytosol. β-sheet #1 in ECL2 is present in the three A34 receptors. The ECL2 in A34β1/β2 receptors had an additional two-stranded β-sheet, designated β#2. The disulfide bridge that anchors the ECL2 β#1 to TMM6 are shown as ball and sticks (circle), which are located between C85 and C164 in Gl-CFRC-A34α2; between C352 and C273 in Gl-CFRC-A34β1; and between C208 and C287 in Gl-CFRC-A34β2.

The structures of the *G. lateralis* A24α/β1, A34α2, and A34β1/β2 CFRCs were modeled using AlphaFold2. Gl-CFRC-A2α1 and Gl-CFRC-A24β2 were not included in the modeling, as they were partial sequences with incomplete open reading frames (**Table 2**; **Supplementary Data 2**). Each full-length CFRC sequence consisted of a single polypeptide with seven transmembrane domains and a topology with the N-terminus oriented on the extracellular surface and the C-terminus on the intracellular surface (**Figures 8**, **9**; depicted as ribbon diagrams on the left for each receptor). The AlphaFold2 models of these GPCRs were evaluated using a per-residue confidence score (pLDDT) between 0 and 100 and the results shown in the structures on the right for each receptor (**Figures 8**, **9**). Regions corresponding to α-helical transmembrane domains showed very high confidence (pLDDT > 90), representing over one-third of the three-dimensional structure. The ECL2 β-sheets had very high (pLDDT > 90) to confident (90 > pLDDT > 70) scores. The other parts of the models, such as ECL3 and intracellular loops, were mostly represented as unresolved loops with low (70 > pLDDT > 50) scores. It should be noted that AlphaFold2 introduces bias in modeling TMM6 and ICL3 regions. This is attributed to the fact that most currently available high-resolution structures were obtained with engineered GPCRs that lacked major portions of the ICL3 and the C-terminal domains (37, 73).

ECL2 and ECL3 form the ligand-binding region of GPCRs (33). The Gl-A24α/β1 and A34α2 ECL2 regions have a single two-stranded β-sheet with a conserved cysteine (C) forming a disulfide bridge to a conserved cysteine (C) in TMM6 (**Figures 8A, B**; **9A**). The ECL2 regions of Gl-A34β1/β2 had a second two-stranded β-sheet formed from the 16 or 17 amino acid insertions in the two sequences (**Figures 9B, C**). This feature distinguished CFRC-A34β1 and -A34β2 from all the other crustacean GPCRs. Consistent with AlphaFold2 models, RosettaFold also predicted two two-stranded β-sheets in the ECL2 of Gl-A34β1/β2 (data not shown).

**Figure 10:**
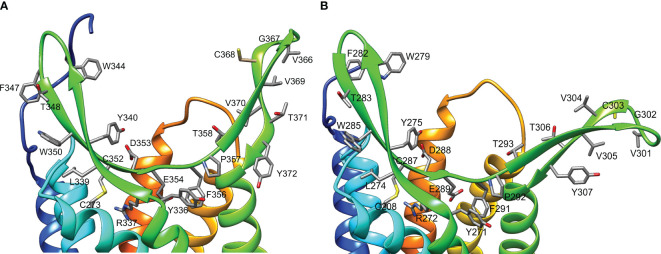
Structure of the ligand-binding region of *G. lateralis* A34β1 **(A)** and A34β2 **(B)** CFRCs. Ribbon diagrams include the side chains of conserved amino acids in the β-sheets of the ECL2 region (YRxxYxxxWxxFTxWxCDExFP in β-sheet #1; VGCVVTY in β-sheet #2). In A34β1, C352 in β-sheet #1 formed a disulfide bridge with C273 in TMM6. In A34β2, C287 in β-sheet #1 formed a disulfide bridge with C208 in TMM6. The distal region of β-sheet #2 had a conserved cysteine located at position #368 in A34β1 and at position #303 in A34β2. Two acidic residues (D353 and E354 in A34β1 and D288 and E289 in A34β2) were located at the bottom of the pocket formed by the β-sheets. Images were produced using the Chimera package. Intracellular regions are not shown for clarity.

Multiple sequence alignment of ECL2 sequences of decapod CFRC-A34β1/β2 identified a conserved motif of 41-42 amino acid residues in A34β1 and 40-41 amino acid residues in A34β2 (**Table 5**; **Figure 6**). Restricting the alignment to the A34β1 and A34β2 sequences from brachyuran species (**Table 4**), two consensus sequences were identified within the motif. The conserved residues were included in structural models of the Gl-A34β1/β2 ligand-binding domain (**Figure 10**). The YRxxYxxxWxNFTxWxCxExFP brachyuran consensus sequence included the cysteine (C) in β-sheet #1, while the CxVxxDAK sequence included the cysteine (C) in β-sheet #2 (**Table 5**). A notable feature of both receptors was that ECL2 had conserved hydrophobic residues projecting from both β-sheets (**Figure 10**). The sequences in the ECL2 motif were highly conserved in the Gl-CFRC-A34β1 and -A34β2 proteins, with YRxxYxxxWxxFTxWxCDExFP and VGCVVTY residues identified (**Figure 10**, compare A and B). There were two acidic residues in the first motif (D353 and E354 in A34β1 and D288 and E289 in A34β2) located in the center of the binding pocket formed by the two β-sheets (**Figure 10**).

## Discussion

Phylogenetic analysis using CrusTome identified homologs of insect ITP GPCRs in crustacean taxa, including copepods, isopods, amphipods, euphausiids, and decapods. They were organized into three large clades named after the *B. mori* BNGR-A2, -A24, and -A34 receptors (**Figure 1**). As members of the GPCR family are hypothesized to mediate CHH family neuropeptide activity, these homologs are designated CHH family receptor candidates, or CFRCs. In decapods, MIH, CHH (eyestalk and pericardial organ isoforms), GIH, and MOIH are among the potential ligands (5). An A2, A24, and A34 classification nomenclature, based on subclades of Pancrustacea sequences, is proposed to provide a consistent framework for naming CFRC sequences. The A2α, A24α, and A34α subclades had sequences from both hexapods and crustaceans, whereas the A2β, A24β, A34α1/2, and A34β subclades had sequences from crustaceans only (**Figures 2**-**4**).

Utilization of the CrusTome database has greatly expanded the number of decapod CFRCs. Previous studies identified a *BNGR-A24* homolog in *P. clarkii* and *BMGR-A34* homologs in *C. maenas*, *P. clarkii*, *S. verreauxi*, *G. lateralis*, *S. paramamosain*, *P. trituberculatus*, *E. sinensis*, *H. americanus*, *C. sapidus*, and *P. argus*, but no homologs in the *BNGR-A2* clade (17, 19, 21–24). One hundred and seventeen sequences from 37 decapod species were organized into 11 CFRC subclades (**Table 4**). This includes the 23 published CFRC sequences and 18 newly-identified sequences in seven decapod species (**Table 1**). The additional sequences, except *Cm-CFRC-A34β3*, were in the A2 and A24 clades (**Table 1**). It should be noted that no new sequences were identified for *P. trituberculatus* and only one new sequence (*Sv-CHHR3*), provided by T. Ventura, was identified in *S. verreauxi* (**Table 1**), as transcriptomic data from both species were not included in the current version of CrusTome (41). The contigs were assigned to ten of the 11 decapod CFRC subclades (**Table 1**). *CFRC-A24β3* was not expressed in the seven species; it appears to be relatively rare, as it was found in only three decapod species (**Table 4**). None of the 37 decapod species expressed sequences for all 11 CFRC subclades; the number ranged from one in *N. denticulata* and two other species to nine in *P. clarkii* (**Tables 1**, **4**). The absence of sequences in the transcriptomes may be due to the tissue source, low expression level, and/or sequencing depth.

Activation of vertebrate Class A GPCRs involves three conserved motifs located in the transmembrane domain and cytoplasmic region, forming an activation pathway that transmits ligand binding to G proteins (30). These motifs are an E/DRY motif located at the boundary of TMM3 and ICL2; a CWxP motif and a conserved phenylalanine (F) that interacts with the tryptophan (W) in TMM6; and an NPxxY motif located at the boundary between TMM7 and the C-terminus (29, 30, 36, 74). The CWxP motif and a conserved phenylalanine (F) were retained in all the CFRCs (**Figure 6**; reference alignment positions #585 to #591), which supports its critical role in receptor activation (36). The NPxxY motif was also present in all the CFRCs (**Supplementary Data 5**). Upon activation, the tyrosine (Y) in the NPxxY motif interacts with hydrophobic residues between TMM6 and TMM7 to stabilize conformational changes in the transmembrane domain (29). The arginine (R) in the E/DRY motif acts as a microswitch; upon receptor activation, it interacts with a conserved tyrosine (Y) located at the boundary of TMM5 and ICL3 and participates in the binding of G proteins (29). The tyrosine was present in all CFRCs (**Figure 6**; reference alignment position #534). However, the DRY sequence in the Gl-CFRC-A24 sequences was replaced with GRF in the Gl-CFRC-A34 sequences (**Supplementary Data 5**). The conservation of the arginine (R) and tyrosine (Y) residues suggests that the activation mechanism in the A24 and A34 receptors is retained. However, the replacements of the aspartate (D) with glycine (G) and the tyrosine (Y) with phenylalanine (F) suggest that the CFRC-A24 and -A34 receptors differ in G protein binding affinity and/or specificity.

The expansion and diversity of CFRCs reflect the large variety of arthropod neuropeptides that bind GPCRs (11, 17, 24, 25, 75, 76). The CHH neuropeptide superfamily is unique to arthropods, but it is greatly expanded in crustaceans. ITPs occur in insects, whereas CHH, MIH, MOIH, and GIH occur only in decapods (8, 22, 77–79). These large neuropeptides have a unique compact core structure consisting of four or five α-helical regions and stabilized by three intramolecular disulfide bridges (3, 9, 12, 13). However, differences in N- and C-terminal sequences, chemical modifications, and distribution of surface amino acid residues confer ligand/receptor binding affinity and specificity. The N- and C-termini of MIH and CHH are essential for biological activity and likely contribute to their binding to distinct high-affinity membrane receptors (8, 9, 80).

The differences in the consensus sequences of brachyuran CHH and MIH peptides (**Table 6**) raise the possibility that co-evolutionary processes have resulted in complementary changes in the receptor regions involved in binding and/or in discriminating structurally similar neuropeptides. In *G. lateralis*, the N-terminal regions of MIH and CHH were highly divergent (**Table 6**; **Figure 7**). This suggests that the N-terminal sequences of these neuropeptides contribute to interactions with the ECL2 and ECL3 regions of Gl-CFRC-A24α, Gl-CFRC-A24β1, and Gl-CFRC-A34β1/β2. Cryo-electron microscopy of human chemokine/GPCR complexes shed light on the peptide-binding mechanism in CFRCs (28, 33). Initially, the chemokine core binds to the N-terminus and ECL2 of the receptor; these regions determine GPCR ligand specificity and affinity (28, 29, 33). This is followed by interactions between the flexible N-terminus of the chemokine with negatively charged residues located on the extracellular regions of the transmembrane core (29, 35). Most of the brachyuran A34β1 and A34β2 proteins had two acidic residues located at the bottom of binding pocket formed by the two β-sheets, suggesting that there are similar interactions between MIH/CHH ligands with these CFRCs (**Figures 6**, **10**). In the structures of chemokine ligands bound to their Class A GPCRs, both in the presence of Gi/o proteins, the peptide or protein ligand binds to extracellular pockets formed by ECL2, ECL3, and the transmembrane core. Specifically, in the CC motif ligand 20 (CCL20)/CC motif receptor 6 (CCR6) complex, the N-terminus of CCL20 interacts with the extracellular crevice of the seven transmembrane core of CCR6, forming crucial interactions with ECL2 and the receptor’s N-terminus (81). Likewise, in the CCL15/CCR1 complex, the N-terminal region and 30s loop of CCL15 are positioned within the seven transmembrane pocket of CCR1, making contact with ECL2 and ECL3, as well as with TMM5 and TMM6 through an extensive network of hydrogen bonds and hydrophobic interactions (82). This suggests that the N-terminal sequences of CHH superfamily neuropeptides may determine binding to specific CFRC subclades, which differ in the ECL2 and ECL3 regions.

A common approach taken for the study of ligand/receptor evolution compares receptors and ligands in non-model organisms, using knowledge from well-studied models, such as mammals and a limited number of arthropods (e.g., *Bombyx, Daphnia*, and *Drosophila)*. However, these pair-to-pair comparisons between classical models and non-model organisms have limitations (83). The approach taken here, which involves comparisons of multiple related organisms in a coherent phylogenetic framework, can provide more accurate reconstructions of ligand/receptor evolution (35). Incorporating hormone signaling mechanisms within an interspecific context can inform biological principles that guide species diversification, adaptation, and survival (84). Thus, analyzing these peptides and their GPCR partners within an evolutionary context provides additional insights regarding gene duplication and functional diversification across invertebrates and Arthropoda, which in turn significantly expands our understanding of the molecular evolution of neuropeptide signaling systems and the co-evolutionary dynamics of peptide-receptor pairs.

Phylogenetic analysis assisted with narrowing the number of potential CHH superfamily receptors in decapods. MIH, CHH, GIH, and MOIH are unique to decapods (5, 8). Assuming ligand/receptor co-evolution, it follows that peptide ligands unique to decapods would bind to receptors that would also be unique to decapods. Of the 11 CFRCs identified in decapods (**Table 4**), eight were decapod-only. The CFRC-A2 subclades were not restricted to decapods. The A2α1 subclade included decapods, hexapods, euphausiids, and peracarids, whereas the A2β subclade included decapods and copepods (**Figure 2**). Three of the four A24 subclades (A24β1/β2/β3) were restricted to decapods; A24α included decapods, copepods, and hexapods (**Figure 3**). All five A34 subclades (A34α1/α2 and A34 β1/β2/β3) were restricted to decapods (**Figure 4**). The three A24 subclades and the five A34 subclades varied in the sequence and structure of the ECL2 and ECL3 regions, suggesting that they bind different ligands (**Figures 5**, **6**, **8**, and **9**). Among the three extracellular loops, ECL2 stands out as the longest and most diverse in terms of sequence length, composition, and structural shape (27, 28, 32, 34, 35). In human Class A GPCRs, the ECL2 region is organized into seven clusters with the peptide and protein GPCRs forming the largest cluster (32). The ECL2 of the Gl-CFRC-A24 and -A34 models exhibited a β-sheet structure, similar to the majority of Class A human GPCRs, and also featured a conserved cysteine (C) that serves as an anchor, tethering ECL2 to the helical bundle in TMM3 (**Figure 8**, **9**) (26, 27, 32, 34, 35, 82). This anchoring may have implications for ligand binding and receptor function, suggesting a potentially crucial role for ECL2 in the context of ligand/receptor interactions.

The CRFC-A34β subclades appear to be the best receptor candidates for MIH and other decapod CHH family neuropeptides. Compared to the A24 clade, the A34 clade showed the greatest expansion and diversification, potentially producing CFRCs with ECL2 and ECL3 regions that can distinguish CHH family neuropeptides (**Figures 4**, **6**) (9). The similarity in structures of the CFRC-A34 subclades (**Figure 9**) with chemokine receptors suggests that the ECL2 forms a lid-like structure over the binding pocket. Interactions between the surface amino acid residues on the neuropeptide with conserved residues projecting from the ECL2 β-sheets (**Figures 7**, **10**) likely contribute to peptide-receptor specificity. As GPCRs often bind to multiple ligands, and vice versa, disentangling the precise mechanisms by which these receptors modulate their binding affinities and specificities becomes of utmost importance to identify optimal ligand-receptor pairs (28). The sequence identity of the ECL2 motif in Gl-CFRC-A34β1 and -A34β2 suggest that the receptors bind the same ligand(s) (**Figure 10**). Peptide binding by GPCRs is of a dynamic nature that involves conformational changes of receptor and ligand structures (28–30, 33, 75), processes which cannot be easily simulated *in silico* when protein crystal structures are not available, as is the case for crustacean GPCRs. These limitations further highlight the significance of integrative approaches within an evolutionary context for the study of non-traditional model organisms.

An aim of this study was to identify potential MIH receptor candidates in two brachyuran species, *G. lateralis* and *C. maenas*. Both species are important models for understanding the endocrine control of molting (2–4, 6, 85–91). Moreover, *C. maenas* is an invasive species aided by anthropogenic range expansion to temperate coastal regions globally (92). Its rapid growth rate, in which the animal nearly doubles its size due to drinking of large quantities of sea water at each ecdysis, has contributed to its success (93, 94). Contigs encoding CFRC-A24β1/β2 in *C. maenas* and *G. lateralis*, A34α2 in *G. lateralis*, A34β1/β2 in *C. maenas* and *G. lateralis*, and A34β3 in *C. maenas* (**Tables 2** and **3**) should be considered putative receptors for CHH superfamily neuropeptides, including MIH, GIH, MOIH, and the eyestalk ganglia and pericardial CHH isoforms generated by alternative splicing (5, 10, 80). As CFRC-A34β1 and -A34β2 are expressed in the YOs of both species (**Tables 2**, **3**) (17, 23), they should be considered candidates for the MIH receptor. However, deorphanizing CFRCs requires functional assays, such as *in vitro* receptor activation assays using recombinant neuropeptide with CFRCs expressed in a cell reporter system and/or *in vivo* studies using double-stranded RNA to knock down receptor expression.

Although none of the receptors for CHH superfamily neuropeptides has been identified in decapods, the identity of the MIH receptor(s) has received the most attention (5, 6). As functional assays are laborious and time consuming, it is useful to consider criteria in prioritizing CFRCs for testing:

1. The MIH receptor(s) should be preferentially expressed in the YO (3). In *G. lateralis*, *Gl-CFRC-A34α2*, *-A34β1*, and *-A34β2* were expressed in the YO and ESG transcriptomes [**Table 2** (17)]. By contrast, contigs encoding *Gl-CFRC-A2α1*, *-A24α*, *-A24β1*, and *-A24β2* were present in the ESG transcriptome (**Table 2**). Endpoint RT-PCR showed qualitative differences in tissue expression of *Gl-CFRC-A34α2* and *Gl-CFRC-A34β1*. *Gl-CFRC-A34α2* (formerly *Gl-CHHR12*) is expressed in YO, hindgut, hepatopancreas, and testis, whereas *Gl-CFRC-A34β1* (formerly *Gl-CHHRA9*) is expressed in YO, eyestalk ganglia, gill, heart, midgut, and thoracic ganglion (17). The tissue expression of *Gl-CFRCβ2* was not determined (17). In *C. maenas*, only *CFRC-A24β1* and *CFRC-A34β1* were present in the YO transcriptome [**Table 3** (23)]. Although differential tissue expression of CFRCs is reported for *E. sinensis*, *S. paramamosain*, and *P. trituberculatus*, expression in the YO was not included in the analysis (22).
2. CFRC expression may change over the molt cycle, reflecting the decrease in sensitivity of the YO to MIH during mid- and late premolt (2). In *G. lateralis* YO, MIH signaling genes, such as adenylyl cyclases, protein kinase A, nitric oxide synthase, calcineurin, and protein kinase G, are down-regulated during premolt (43). *Gl-CFRC-A34α2*, *-A34β1*, and *-A34β2* show different patterns of relative expression over the molt cycle, with *Gl-CFRC-A34β1* showing a pattern consistent with the down-regulation of other MIH signaling genes. Expression of *Gl-CFRC-A34β1* (formerly *Gl-GPCR-A9*) is highest at intermolt, decreases during premolt, and is lowest at postmolt (17). Expression of *Gl-CFRC-A34β2* (formerly *Gl-GPCR-A10*) is highest during premolt and is lowest at postmolt (17). Expression of *Gl-CFRC-A34α2* (formerly *Gl-GPCR-A12*) is low at intermolt, early premolt, and mid-premolt, highest at late premolt, and lowest at postmolt (17). It is worth noting that GPCRs are generally expressed at very low levels (17), suggesting that any change in expression may not translate to meaningful changes in the number of receptors in the membrane. For example, binding of radiolabeled Cm-MIH to *C. maenas* YO membrane preparations is not affected by molt stage (95).
3. The high conservation of brachyuran MIH and CHH sequences and structure, as well as biological activity, suggests a strong ligand/receptor co-evolution. For example, an antibody raised against a conserved N-terminal peptide sequence in Gl-MIH (amino acid residues #7 to #20 in the mature peptide) cross-reacts with Cm-MIH (86). *G. lateralis* rMIH and the eyestalk rCHH isoform inhibit ecdysteroid secretion in YOs from *C. maenas* (71, 96). The actions of MIH and CHH in *C. maenas* are mediated by distinct high-affinity receptors in the YO membrane (97). Moreover, similar concentrations of MIHs from two other brachyuran species, *Necora puber* and *Cancer pagurus*, can displace radiolabeled Cm-MIH from *C. maenas* YO membranes (97).

## Conclusions

The MIH receptor is a critical component of the signal transduction pathway that regulates YO ecdysteroid synthesis (2, 6). Assuming that the MIH receptor is a Class A GPCR, the challenge has been identifying potential candidates from among the large number of YO Class A GPCRs for functional analysis (6, 17, 23). Phylogenetic analysis has been used to characterize homologs of *Bombyx* ITP GPCRs in decapod transcriptomes. Previous studies have used this approach, mostly identifying homologs in the A34 clade (17, 19, 21–24). Phylogenetic analysis with the CrusTome database greatly expanded the number of CFRC homologs in the Crustacea, which were organized into a classification nomenclature corresponding to the *Bombyx* ITP BNGR-A2, -A24, and -A34 phylogeny (**Figure 1**, **Table 4**, and **Supplementary Data 2**). This nomenclature provides a framework for characterizing new homologs/orthologs as more transcriptomic data become available. A total of 11 CFRC subclades were identified in decapod crustaceans, although none of the 37 decapod species expressed all 11 (**Table 4**). This suggests that expression of certain CFRCs is restricted to specific tissues, enabling target tissues to respond to neuropeptides that control physiological processes, such as molting, reproduction, metabolism, ion and water balance, and responses to environmental stress (3, 5, 10). Analysis of the ECL2 and ECL3 regions, which mediate ligand binding, identified motifs that can be used to distinguish members of the A2, A24, and A34 clades and subclades (**Table 5**; **Figures 5**, **6**). Structural modeling of the *G. lateralis* CFRCs showed that the ECL2 of A34β1 and A34β2 had a second β-sheet not found in hexapod and other crustacean GPCRs. The two β-sheets form a deep pocket on the extracellular surface of the receptor to accommodate large neuropeptides, such as CHH and MIH. Conserved residues in both β-sheets may stabilize neuropeptide binding with the receptor. These studies, in concert with earlier YO expression analyses, support prioritizing the A34β CFRC subclades as potential MIH receptor(s) for functional assays and structural modeling simulations of ligand/receptor binding.

## Data availability statement

The datasets presented in this study can be found in online repositories. The names of the repository/repositories and accession number(s) can be found in the article/**Supplementary Material**.

## Ethics statement

The manuscript presents research on animals that do not require ethical approval for their study.

## Author contributions

MK: Conceptualization, Data curation, Formal analysis, Investigation, Methodology, Resources, Visualization, Writing – review & editing. JP: Data curation, Formal analysis, Investigation, Methodology, Resources, Visualization, Writing – review & editing, Writing – original draft. NG: Conceptualization, Data curation, Formal analysis, Investigation, Methodology, Resources, Visualization, Writing – original draft, Writing – review & editing. LJ: Investigation, Writing – review & editing, Formal analysis. DD: Funding acquisition, Investigation, Project administration, Writing – review & editing. TV: Conceptualization, Formal analysis, Investigation, Writing – review & editing. DM: Conceptualization, Funding acquisition, Investigation, Project administration, Supervision, Validation, Writing – original draft, Writing – review & editing.

## Supplementary material

Supplementary Data Sheet 1
*G. lateralis* ESG transcriptome data set.

Supplementary Data Sheet 2 Classification and sequence data table.

Supplementary Data Sheet 3 Sequences employed for structural modeling.

Supplementary Data Sheet 4 Alignment and phylogenetic tree files.

Supplementary Data Sheet 5 CFRC and MIH/CHH alignment figures.

Supplementary Data Sheet 6 Coordinates of the AlphaFold2 models (-) in PDB format.
