# Supplementary material for: In silico analysis of crustacean hyperglycemic hormone family G protein-coupled receptor candidates
Source: Front Endocrinol (Lausanne). 2024 Jan 9;14:1322800. doi: 10.3389/fendo.2023.1322800 (PMC10828670; doi:10.3389/fendo.2023.1322800)
Supplement: Supplementary Data Sheet 1 — G. lateralis ESG transcriptome data set. [file DataSheet_1.zip › Supplementary Data/SuppData1.docx]

ESG transcriptome can be found at:

Kozma, M. T., Perez-Moreno, J. L., Durica, D. S., & Mykles, D. L. (2023).

Gecarcinus lateralis - Eyestalk Ganglia Transcriptome (1.0) [Data set]. Zenodo.

https://doi.org/10.5281/zenodo.7987266
