# Supplementary material for: In silico analysis of crustacean hyperglycemic hormone family G protein-coupled receptor candidates
Source: Front Endocrinol (Lausanne). 2024 Jan 9;14:1322800. doi: 10.3389/fendo.2023.1322800 (PMC10828670; doi:10.3389/fendo.2023.1322800)
Supplement: Supplementary Data Sheet 1 — G. lateralis ESG transcriptome data set. [file DataSheet_1.zip › Supplementary Data/SuppData3.docx]

Methods: UCSF ChimeraX version 1.5, a free multi-platform molecular modelling program developed by the Resource for Biocomputing, Visualization, and Informatics at the University of California, San Francisco, was used to model and visualise 3D structures. The sequences of receptors were truncated at the N- and C-terminal cytoplasmic domain due to intrinsic disorder. The truncated sequences (reported in Table below) were subjected to AlphaFold2 using the ChimeraX interface to submit 3D structure prediction to run at Google Colab. The predicted structures were energy minimised and the best model out of five was selected for further analysis. Other research groups have optimised AlphaFold2 to predict either active or inactive states of GPCRs. We didn’t use these advanced methods due to the absence of 3D structures of GPCRS in crustaceans.

Figure 1 The ribbon diagram representing MIH and CHH is shown with colours ranging from blue for the N terminus to red for the C terminus. The MIH has an additional turn at the N terminus. Both MIH and CHH have three conserved disulphide bridges shown in stick representation.

Figure 2 The ribbon diagram representing receptors A34a and A34b is shown with colours ranging from blue for the N terminus to red for the C terminus. All receptors show a common topology composed of seven transmembrane α helices connected by three extracellular and three intracellular loops. The N terminus is in the extracellular space, and the C terminus is in the cytosol. The disulphide bonds are shown as ball and sticks. In A34 receptors, disulphide bonds between C352 and C273 (A34a) and C208 and C287 (A34b) are conserved. Molecular graphics images were produced using the Chimera package (UCSF).

Figure 3 The ribbon diagram representing receptors A24a and A24b is shown with colours ranging from blue for the N terminus to red for the C terminus. All receptors show a common topology composed of seven transmembrane α helices connected by three extracellular and three intracellular loops. The N terminus is in the extracellular space, and the C terminus is in the cytosol. The disulphide bonds are shown as ball and sticks. In A34 receptors, disulphide bonds between C148 and C227 (A24a) and C178and C258 (A34b) are conserved. Molecular graphics images were produced using the Chimera package (UCSF).

| Name | Sequences | Sequences used in modelling |
| --- | --- | --- |
| MIH | >ABF06632.1 molt-inhibiting hormone precursor [Gecarcinus lateralis]  MMSRAESRFASQRTWLVAVVVLAVLWSIGVQRAAAAVINDECPNVIGNRDIFKKVDWICEDCANIFRIDG  LATLCRKNCFRNIDFLWCVYASERQAEKDELTRYVSILRAGSV | AVINDECPNVIGNRDIFKKVDWICEDCANIFRIDG  LATLCRKNCFRNIDFLWCVYASERQAEKDELTRYVSILRAGSV |
| CHH | >ABF48652.1 crustacean hyperglycemic hormone A [Gecarcinus lateralis]  MTSRMTSAMALVVVAVCASLYSLPHAHARSADGFGRMERLLSSLRGSAESSGALGELRGAGEASAAHPLEKRQIYDRSCKGVYDRSLFNKLEHVCDDCYNLYRTSFVYSSCRENCYSNLVFRQCMEDLLLMDVFDEYAKAVQVVGRKKK | QIYDRSCKGVYDRSLFNKLEHVCDDCYNLYRTSFVYSSCRENCYSNLVFRQCMEDLLLMDVFDEYAKAVQVVG |
| A34a | >Glat_CHHrA34a_ESG_GeclatEVm004216t2_Gl_CHHRA9 type=protein; aalen=453,51%,partial5-utrpoor; clen=2657  QQEEHGNQEEGASRRRRRRRRRRRGIVLSYGLQCVLEPPPPPPSPPLLLLLLPTPLPPPSLPPPPPPPPHPS  QQEEHGNQEEGASRRRRRRRRRRRGIVLSYGLQCVLEPPPPPPSPPLLLLLLPTPLPPPSLPPPPPPPPHPS  TIMASTSLPAISYSPMEDLVNLSYACQQDPRITTNFSQYEYTYRMKTWVPFTWREVLKVVAYLVVFLVSLIG  NLLVILVVCYNRHMRTSTNQYLVNLAAADLLVTLVCMWVHIVRHLSYPHYVLPALVCKLDGFVQTTTLLASV  LTLTVISVGRFVAVMFPLHARTSPDRANRVIATVWIASALLACPTLFYRELYSIEWANFTTWQCDEFFPTER  EYVKDVGCVVTYDAKQLFYTILNIALYFLPVAIMIINYSLVVWTLWGAKQPGEHHSAATRNMATRAKKRVVK  MVTVVLVVFVICWTPLQTLILYSSFSQEDHLPEWFSTLEFAAYFVAYSNSALNPITCPRPPPPPPQERRFTG  QAERREAQHSGVILVGGQRGA | SQYEYTYRMKTWVPFTWREVLKVVAYLVVFLVSLIGNLLVILVVCYNRHMRTSTNQYLVNLAAADLLVTLVCMWVHIVRHLSYPHYVLPALVCKLDGFVQTTTLLASVLTLTVISVGRFVAVMFPLHARTSPDRANRVIATVWIASALLACPTLFYRELYSIEWANFTTWQCDEFFPTEREYVKDVGCVVTYDAKQLFYTILNIALYFLPVAIMIINYSLVVWTLWGAKQPGEHHSAATRNMATRAKKRVVKMVTVVLVVFVICWTPLQTLILYSSFSQEDHLPEWFSTLEFAAYFVAYSNSALNPITCPRPPPPPPQERRFTG |
| A34b | >Glat_CHHrA34b_ESG_GeclatEVm002817t1 type=protein; aalen=688,60%,partial3; clen=3435  MFEVSDATTGVEGTEAGVWTNESAGEIWSGGGAVTTTNTTSTFSSTFASSSSPLSHLLHMALTSTSSPSPSS  ASSFASSTDLPLFSTTYYMDDAANRSMLMPNSCDVNPLLFTDFAQYEYPYRTDTWIPITWREVLKLVAYIIT  FLVSIAGNILVILVVRYNRNMRSSTNQYLVNLAVADLLVTLVCKWVHLVRHLSYPHYVLPALVCKLDGFVQG  TALVASVFTLTVISIGRFVAIMFPLHARTSPDRAIRVIAAVWIASALISSPMLFYRELYSTEWSNFTAWNCD  EAFPTERKFVKGVGCVVTYDAKQLFYVIFTITCYFLPVTIMLVNYSLIVWKLWGAQQPGEQHQQQVAATRNM  QRVVRMVTVVLVVFVICWTPLQSLILYTTFSNDEHVPEWLSWLEFTAYFVAHSNSALNPIIYCGFNANFRQG  LVALLTCRQSRSGSRTYYPRSWRGLTGTRESITGYSGPEPAVVLDMGGSVRHSRLTQKSSSSSLCGRLVYTG  SARELRHDNHHHHHLCDPLNTSGITSISSINSNGGSGSGGNSITSNRNLSLRTNSTSMRQGYRSTNRSSDQQ  ELTLAQRTFVRASYMGDNGRLDALRASTSSSSYTHRGGGRVGGGLDKEDEYGCSCCGGGKGRKGGGGRGGGG  GGGGTGIGGRGSGGGTRSLWGDRGGGGGGTGIGGRGSGGG | YRTDTWIPITWREVLKLVAYIIT  FLVSIAGNILVILVVRYNRNMRSSTNQYLVNLAVADLLVTLVCKWVHLVRHLSYPHYVLPALVCKLDGFVQG  TALVASVFTLTVISIGRFVAIMFPLHARTSPDRAIRVIAAVWIASALISSPMLFYRELYSTEWSNFTAWNCD  EAFPTERKFVKGVGCVVTYDAKQLFYVIFTITCYFLPVTIMLVNYSLIVWKLWGAQQPGEQHQQQVAATRNM  QRVVRMVTVVLVVFVICWTPLQSLILYTTFSNDEHVPEWLSWLEFTAYFVAHSNSALNPIIYCGFNANFRQG  LVALLTCRQSRSGSR |
| A24a | >Glat_CHHrA24a_ESG_GeclatEVm005146t1_Gl_CHHR3 type=protein; aalen=476,56%,complete-utrpoor; clen=2533  MELIAEPIMDLEGPGNTTALMATHDWWNSTDVDVDVSVNCSRTFCPVTNVTNSTCVECPLLDDEGNKYNLPW  WNELIWYVVFMGMVIVATGGNTIVIWIVLADRRMRTVTNIFLVNLSVADAMVSTLNVVFNFTYMLNLNWRFG  FVYCKISQFVSILSICASVFTLMAISFDRYIAIMHPLRPRMGRKATILIVVWIWVSSVCLSLPNLIYFTTAT  LSYAGGERIVCYAAWPDGDQGESQSEYVHTVVLMVLTYILPLTCMGFTYVRIGLTLWGSRSIGEQTPRQVES  IKSKRKVVKMMIVVVTIFAVCWLPYHMYFILSNLMPEIAHYEFIQETYLAIYWLAMSNSMYNPMIYCWLNNR  FRNGFKKVFSGWLPCITYEGETAEVTRVKTARYSCSGSPETHHRVTYDGSSHISMRNLASSDLSISGSRLPP  VRLCNGANLRRATNGADSASRGYGGTLGPPKHTPYPSVTYQQYV | LLDDEGNKYNLPW  WNELIWYVVFMGMVIVATGGNTIVIWIVLADRRMRTVTNIFLVNLSVADAMVSTLNVVFNFTYMLNLNWRFG  FVYCKISQFVSILSICASVFTLMAISFDRYIAIMHPLRPRMGRKATILIVVWIWVSSVCLSLPNLIYFTTAT  LSYAGGERIVCYAAWPDGDQGESQSEYVHTVVLMVLTYILPLTCMGFTYVRIGLTLWGSRSIGEQTPRQVES  IKSKRKVVKMMIVVVTIFAVCWLPYHMYFILSNLMPEIAHYEFIQETYLAIYWLAMSNSMYNPMIYCWLNNR  FRNGFKKVFSGWLPCITYEGETAE |
| A24b | >Glat_CHHrA24b_ESG_GeclatEVm002823t1 type=protein; aalen=687,78%,complete; clen=2623  MMEEKEKDNGEGAESGGAGEGWPALLFECVLQVWQELNATTDLPAPLNVSHNIYFEHEFYMRLLNLSESGEL  NGTDWEAGGRDRLAQCLEPPPADRPYLLPWWQQLTWTLAFGAMLLVAVGGNAIVMWIVIAHRRMRTVTNYFL  VNLSAADLLMAVFNCIFNFIYMLHSDWPFGAVYCTISNFMANVTIAASVFTLMAISFDRYIAIVRPLKPRMS  KSEARHFIIFIWLSSMSLAVPCLLYSTTVSIRYKNDEIRRGCFLLWPDGKTSISYREYVYNIVFFATTYVLP  MLVMLVSYTLIGCELWGSHSIGELTDRQVSSIKSKRRVVRMFIVIVVVFMLCWLPQQGFFLYQYHNSQVLDS  AHIQHIYLGFYWLAMANAMVNPIIYYWMNARFRSYFREVVLQCSSGRCCCCCSAPSTYLDSPHLARRRHDSI  EHTSRSRSAAGGVTPRFCNKTGGKQHHDNFFIRGLGCAKPLDGGYCPTHLKGEAEWHQMRYLTDKGHLTFQK  PADPLYAHNINGVRKPLNDDPLLAKAGHGDDSWTPPSDPAQPQRPSETPDDLLLVPVLASPPLRLPCPAAPP  IANSHSEKESLGQPSKPPAEDSGIEMKSFPNGNNHVAPRVLLSTDSDPEKALAEPCPAGQGHSHNPAGLVHL  AIEETLMAAVLQSCSSDHIGLDPTSKSEDSMKVSKEIML | PPPADRPYLLPWWQQLTWTLAFGAMLLVAVGGNAIVMWIVIAHRRMRTVTNYFLVNLSAADLLMAVFNCIFNFIYMLHSDWPFGAVYCTISNFMANVTIAASVFTLMAISFDRYIAIVRPLKPRMSKSEARHFIIFIWLSSMSLAVPCLLYSTTVSIRYKNDEIRRGCFLLWPDGKTSISYREYVYNIVFFATTYVLPMLVMLVSYTLIGCELWGSHSIGELTDRQVSSIKSKRRVVRMFIVIVVVFMLCWLPQQGFFLYQYHNSQVLDSAHIQHIYLGFYWLAMANAMVNPIIYYWMNARFRSYFREVVLQCSSGRCC |
