# Supplementary material for: In silico analysis of crustacean hyperglycemic hormone family G protein-coupled receptor candidates
Source: Front Endocrinol (Lausanne). 2024 Jan 9;14:1322800. doi: 10.3389/fendo.2023.1322800 (PMC10828670; doi:10.3389/fendo.2023.1322800)
Supplement: Supplementary Data Sheet 1 — G. lateralis ESG transcriptome data set. [file DataSheet_1.zip › Supplementary Data/SuppData6/Table-sequence compilation.docx]

Table : Full-length CFRC sequences and the sequences truncated at the termini for AlphaFold 2 modelling. The sequences were truncated due to intrinsic disorder of the N-terminal and C-terminal domains.

| Name | Sequence | Sequence used in modelling |
| --- | --- | --- |
| MIH | >ABF06632.1 molt-inhibiting hormone precursor [Gecarcinus lateralis]  MMSRAESRFASQRTWLVAVVVLAVLWSIGVQRAAAAVINDECPNVIGNRDIFKKVDWICEDCANIFRIDGLATLCRKNCFRNIDFLWCVYASERQAEKDELTRYVSILRAGSV | AVINDECPNVIGNRDIFKKVDWICEDCANIFRIDG  LATLCRKNCFRNIDFLWCVYASERQAEKDELTRYVSILRAGSV |
| CHH | >ABF48652.1 crustacean hyperglycemic hormone A [Gecarcinus lateralis]  MTSRMTSAMALVVVAVCASLYSLPHAHARSADGFGRMERLLSSLRGSAESSGALGELRGAGEASAAHPLEKRQIYDRSCKGVYDRSLFNKLEHVCDDCYNLYRTSFVYSSCRENCYSNLVFRQCMEDLLLMDVFDEYAKAVQVVGRKKK | QIYDRSCKGVYDRSLFNKLEHVCDDCYNLYRTSFVYSSCRENCYSNLVFRQCMEDLLLMDVFDEYAKAVQVVG |
| A12/1-499 | >Gl_CHHRA12/1-499  MIVFGERELVCNDTCCQMSSGNVLCFDDYNFTDRANITYPESWELAIRWTYAPLVMLVGVLGNLAIIIILAKNRLLLRTSVNYFILNMSVADLITALAGPIPFTIRDTAYFWVLGETWCYLEGYIQMLVMMVSVTSLATISCDRMMGVVRPFHNHLKAWQSITIMFVIWCVSAALAVPFAVYRIYTIHHWQDLTETTCGEEVNKMHVYWMINMIGLTWLPLFVMIVCYTTIFIYFRTHKFQADRKKEHPAITHLRRRVVLMMFIVVIAFTICWLPFQLLKICNKFFLDEENHFNSEAAEKSHDILLTISHYLIYTNAAINPIIYALMHQTFRRAFRVTFPCFYNQKSAFVLTPGEGRRNYVWSMRSTSTMYNIMSMSRNHISERSRKPKELTSSAVVCSAALAASIAIKPISKESREENSLQDSGQTQLPKSHVVSVQVEQGVSLSNNGPRSPPLGNERYVQGLAPRQISIVSTGALGQLITQVIEEETSSDLESERIL | ANITYPESWELAIRWTYAPLVMLVGVLGNLAIIIILAK  NRLLLRTSVNYFILNMSVADLITALAGPIPFTIRDTAYFWVLGETWCYLEGYIQMLVMMVSVTSLATISCDR  MMGVVRPFHNHLKAWQSITIMFVIWCVSAALAVPFAVYRIYTIHHWQDLTETTCGEEVNKMHVYWMINMIGLTWLPLFVMIVCYTTIFIYFRTHKFQADRKKEHPAITHLRRRVVLMMFIVVIAFTICWLPFQLLKICNKFFLDEENHFNSEAAEKSHDILLTISHYLIYTNAAINPIIYALMHQTFRRAFRVTFPCFYNQKSAFVLTPGEGRRNY |
| Gl-CFRC-A34b1_GeclatEVm004216t2 | >Glat_CHHrA34a_ESG_GeclatEVm004216t2_Gl_CHHRA9 type=protein; aalen=453,51%,partial5-utrpoor; clen=2657  QQEEHGNQEEGASRRRRRRRRRRRGIVLSYGLQCVLEPPPPPPSPPLLLLLLPTPLPPPSLPPPPPPPPHPSQQEEHGNQEEGASRRRRRRRRRRRGIVLSYGLQCVLEPPPPPPSPPLLLLLLPTPLPPPSLPPPPPPPPHPSTIMASTSLPAISYSPMEDLVNLSYACQQDPRITTNFSQYEYTYRMKTWVPFTWREVLKVVAYLVVFLVSLIGNLLVILVVCYNRHMRTSTNQYLVNLAAADLLVTLVCMWVHIVRHLSYPHYVLPALVCKLDGFVQTTTLLASVLTLTVISVGRFVAVMFPLHARTSPDRANRVIATVWIASALLACPTLFYRELYSIEWANFTTWQCDEFFPTEREYVKDVGCVVTYDAKQLFYTILNIALYFLPVAIMIINYSLVVWTLWGAKQPGEHHSAATRNMATRAKKRVVKMVTVVLVVFVICWTPLQTLILYSSFSQEDHLPEWFSTLEFAAYFVAYSNSALNPITCPRPPPPPPQERRFTGQAERREAQHSGVILVGGQRGA | SQYEYTYRMKTWVPFTWREVLKVVAYLVVFLVSLIGNLLVILVVCYNRHMRTSTNQYLVNLAAADLLVTLVCMWVHIVRHLSYPHYVLPALVCKLDGFVQTTTLLASVLTLTVISVGRFVAVMFPLHARTSPDRANRVIATVWIASALLACPTLFYRELYSIEWANFTTWQCDEFFPTEREYVKDVGCVVTYDAKQLFYTILNIALYFLPVAIMIINYSLVVWTLWGAKQPGEHHSAATRNMATRAKKR |
| Gl-CFRC-A34b2_GeclatEVm002817t1 | >Glat_CHHrA34b_ESG_GeclatEVm002817t1 type=protein; aalen=688,60%,partial3; clen=3435  MFEVSDATTGVEGTEAGVWTNESAGEIWSGGGAVTTTNTTSTFSSTFASSSSPLSHLLHMALTSTSSPSPSSASSFASSTDLPLFSTTYYMDDAANRSMLMPNSCDVNPLLFTDFAQYEYPYRTDTWIPITWREVLKLVAYIITFLVSIAGNILVILVVRYNRNMRSSTNQYLVNLAVADLLVTLVCKWVHLVRHLSYPHYVLPALVCKLDGFVQGTALVASVFTLTVISIGRFVAIMFPLHARTSPDRAIRVIAAVWIASALISSPMLFYRELYSTEWSNFTAWNCDEAFPTERKFVKGVGCVVTYDAKQLFYVIFTITCYFLPVTIMLVNYSLIVWKLWGAQQPGEQHQQQVAATRNMQRVVRMVTVVLVVFVICWTPLQSLILYTTFSNDEHVPEWLSWLEFTAYFVAHSNSALNPIIYCGFNANFRQGLVALLTCRQSRSGSRTYYPRSWRGLTGTRESITGYSGPEPAVVLDMGGSVRHSRLTQKSSSSSLCGRLVYTGSARELRHDNHHHHHLCDPLNTSGITSISSINSNGGSGSGGNSITSNRNLSLRTNSTSMRQGYRSTNRSSDQQELTLAQRTFVRASYMGDNGRLDALRASTSSSSYTHRGGGRVGGGLDKEDEYGCSCCGGGKGRKGGGGRGGGGGGGGTGIGGRGSGGGTRSLWGDRGGGGGGTGIGGRGSGGG | YRTDTWIPITWREVLKLVAYIITFLVSIAGNILVILVVRYNRNMRSSTNQYLVNLAVADLLVTLVCKWVHLVRHLSYPHYVLPALVCKLDGFVQGTALVASVFTLTVISIGRFVAIMFPLHARTSPDRAIRVIAAVWIASALISSPMLFYRELYSTEWSNFTAWNCDEAFPTERKFVKGVGCVVTYDAKQLFYVIFTITCYFLPVTIMLVNYSLIVWKLWGAQQPGEQHQQQVAATRNMQRVVRMVTVVLVVFVICWTPLQSLILYTTFSNDEHVPEWLSWLEFTAYFVAHSNSALNPIIYCGFNANFRQGLVALLTCRQSRSGSR |
| Gl-CFRC-A24a_GeclatEVm005146t1 | >Glat_CHHrA24a_ESG_GeclatEVm005146t1_Gl_CHHR3 type=protein; aalen=476,56%,complete-utrpoor; clen=2533  MELIAEPIMDLEGPGNTTALMATHDWWNSTDVDVDVSVNCSRTFCPVTNVTNSTCVECPLLDDEGNKYNLPWWNELIWYVVFMGMVIVATGGNTIVIWIVLADRRMRTVTNIFLVNLSVADAMVSTLNVVFNFTYMLNLNWRFGFVYCKISQFVSILSICASVFTLMAISFDRYIAIMHPLRPRMGRKATILIVVWIWVSSVCLSLPNLIYFTTATLSYAGGERIVCYAAWPDGDQGESQSEYVHTVVLMVLTYILPLTCMGFTYVRIGLTLWGSRSIGEQTPRQVESIKSKRKVVKMMIVVVTIFAVCWLPYHMYFILSNLMPEIAHYEFIQETYLAIYWLAMSNSMYNPMIYCWLNNRFRNGFKKVFSGWLPCITYEGETAEVTRVKTARYSCSGSPETHHRVTYDGSSHISMRNLASSDLSISGSRLPPVRLCNGANLRRATNGADSASRGYGGTLGPPKHTPYPSVTYQQYV | LLDDEGNKYNLPWWNELIWYVVFMGMVIVATGGNTIVIWIVLADRRMRTVTNIFLVNLSVADAMVSTLNVVFNFTYMLNLNWRFGFVYCKISQFVSILSICASVFTLMAISFDRYIAIMHPLRPRMGRKATILIVVWIWVSSVCLSLPNLIYFTTATLSYAGGERIVCYAAWPDGDQGESQSEYVHTVVLMVLTYILPLTCMGFTYVRIGLTLWGSRSIGEQTPRQVESIKSKRKVVKMMIVVVTIFAVCWLPYHMYFILSNLMPEIAHYEFIQETYLAIYWLAMSNSMYNPMIYCWLNNRFRNGFKKVFSGWLPCITYEGETAE |
| Gl-CFRC-A24b1_GeclatEVm002823t1 | >Glat_CHHrA24b_ESG_GeclatEVm002823t1 type=protein; aalen=687,78%,complete; clen=2623  MMEEKEKDNGEGAESGGAGEGWPALLFECVLQVWQELNATTDLPAPLNVSHNIYFEHEFYMRLLNLSESGELNGTDWEAGGRDRLAQCLEPPPADRPYLLPWWQQLTWTLAFGAMLLVAVGGNAIVMWIVIAHRRMRTVTNYFLVNLSAADLLMAVFNCIFNFIYMLHSDWPFGAVYCTISNFMANVTIAASVFTLMAISFDRYIAIVRPLKPRMSKSEARHFIIFIWLSSMSLAVPCLLYSTTVSIRYKNDEIRRGCFLLWPDGKTSISYREYVYNIVFFATTYVLPMLVMLVSYTLIGCELWGSHSIGELTDRQVSSIKSKRRVVRMFIVIVVVFMLCWLPQQGFFLYQYHNSQVLDSAHIQHIYLGFYWLAMANAMVNPIIYYWMNARFRSYFREVVLQCSSGRCCCCCSAPSTYLDSPHLARRRHDSIEHTSRSRSAAGGVTPRFCNKTGGKQHHDNFFIRGLGCAKPLDGGYCPTHLKGEAEWHQMRYLTDKGHLTFQKPADPLYAHNINGVRKPLNDDPLLAKAGHGDDSWTPPSDPAQPQRPSETPDDLLLVPVLASPPLRLPCPAAPPIANSHSEKESLGQPSKPPAEDSGIEMKSFPNGNNHVAPRVLLSTDSDPEKALAEPCPAGQGHSHNPAGLVHLAIEETLMAAVLQSCSSDHIGLDPTSKSEDSMKVSKEIML | PPPADRPYLLPWWQQLTWTLAFGAMLLVAVGGNAIVMWIVIAHRRMRTVTNYFLVNLSAADLLMAVFNCIFNFIYMLHSDWPFGAVYCTISNFMANVTIAASVFTLMAISFDRYIAIVRPLKPRMSKSEARHFIIFIWLSSMSLAVPCLLYSTTVSIRYKNDEIRRGCFLLWPDGKTSISYREYVYNIVFFATTYVLPMLVMLVSYTLIGCELWGSHSIGELTDRQVSSIKSKRRVVRMFIVIVVVFMLCWLPQQGFFLYQYHNSQVLDSAHIQHIYLGFYWLAMANAMVNPIIYYWMNARFRSYFREVVLQCSSGRCC |
