# Supplementary figures and images for: In silico analysis of crustacean hyperglycemic hormone family G protein-coupled receptor candidates
Source: Front Endocrinol (Lausanne). 2024 Jan 9;14:1322800. doi: 10.3389/fendo.2023.1322800 (PMC10828670; doi:10.3389/fendo.2023.1322800)

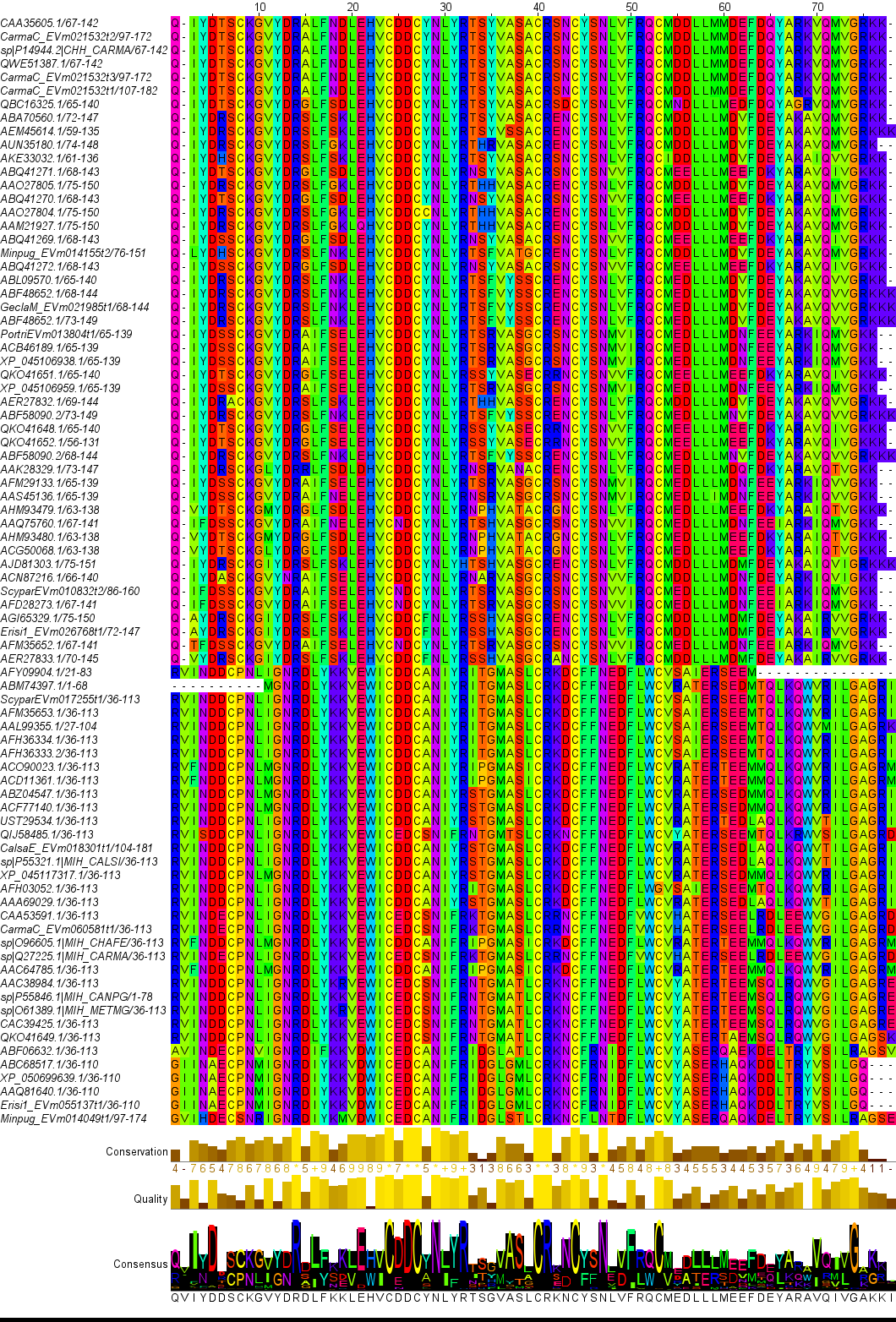

Supplement: Supplementary Data Sheet 1 — G. lateralis ESG transcriptome data set. [file DataSheet_1.zip › Supplementary Data/SuppData5/chh-mih_brachyura.png]

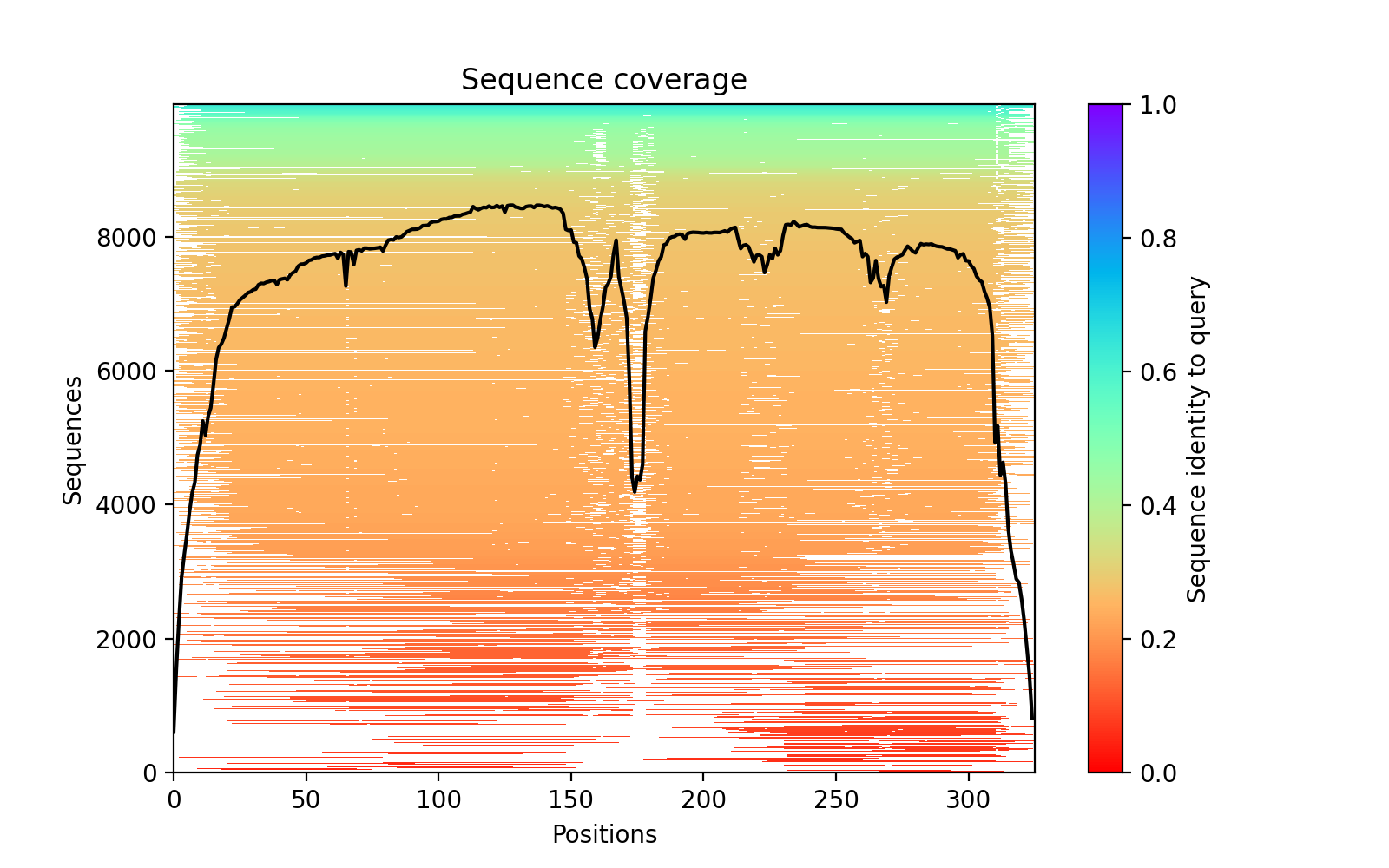

Supplement: Supplementary Data Sheet 1 — G. lateralis ESG transcriptome data set. [file DataSheet_1.zip › Supplementary Data/SuppData6/Gl-A24a/af325_coverage.png]

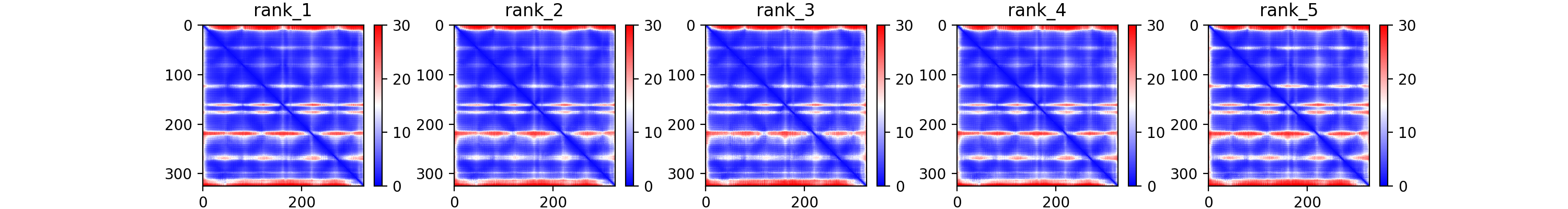

Supplement: Supplementary Data Sheet 1 — G. lateralis ESG transcriptome data set. [file DataSheet_1.zip › Supplementary Data/SuppData6/Gl-A24a/af325_PAE.png]

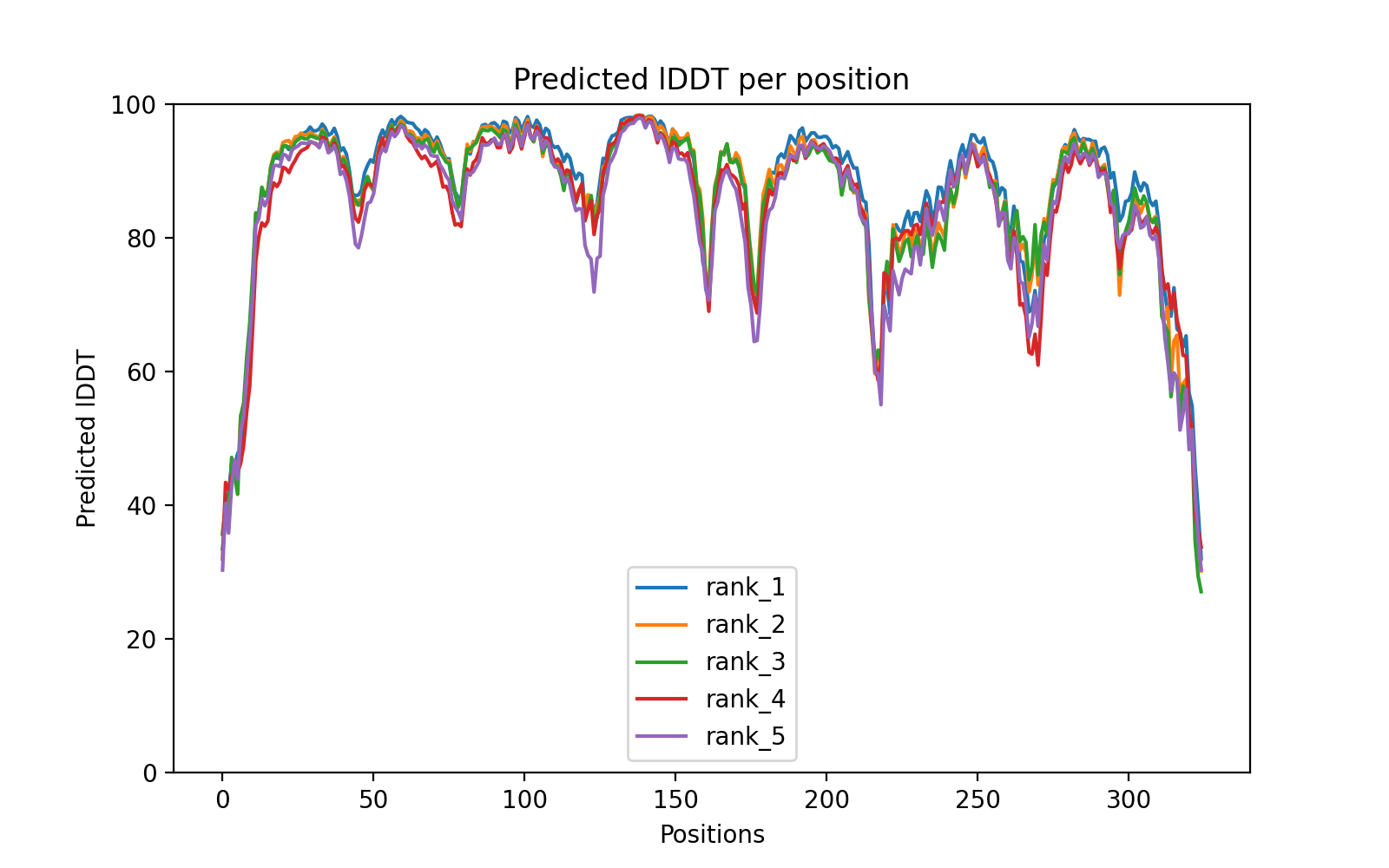

Supplement: Supplementary Data Sheet 1 — G. lateralis ESG transcriptome data set. [file DataSheet_1.zip › Supplementary Data/SuppData6/Gl-A24a/af325_plddt.png]

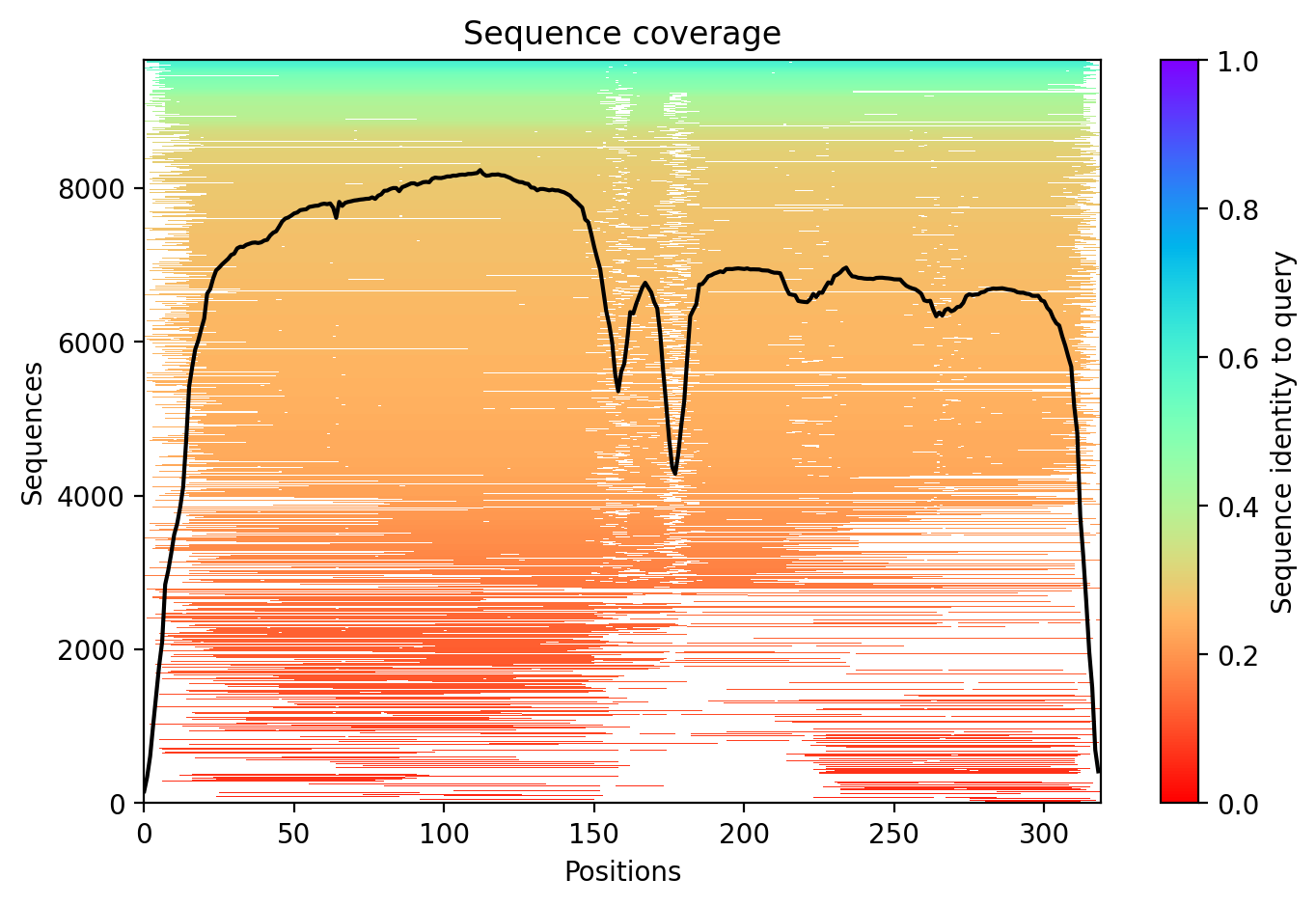

Supplement: Supplementary Data Sheet 1 — G. lateralis ESG transcriptome data set. [file DataSheet_1.zip › Supplementary Data/SuppData6/Gl-A24b1/af319_coverage.png]

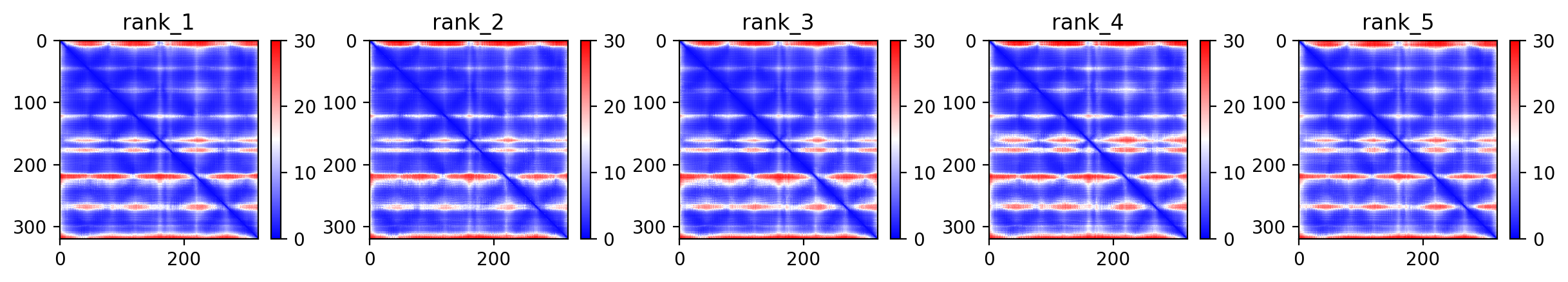

Supplement: Supplementary Data Sheet 1 — G. lateralis ESG transcriptome data set. [file DataSheet_1.zip › Supplementary Data/SuppData6/Gl-A24b1/af319_pae.png]

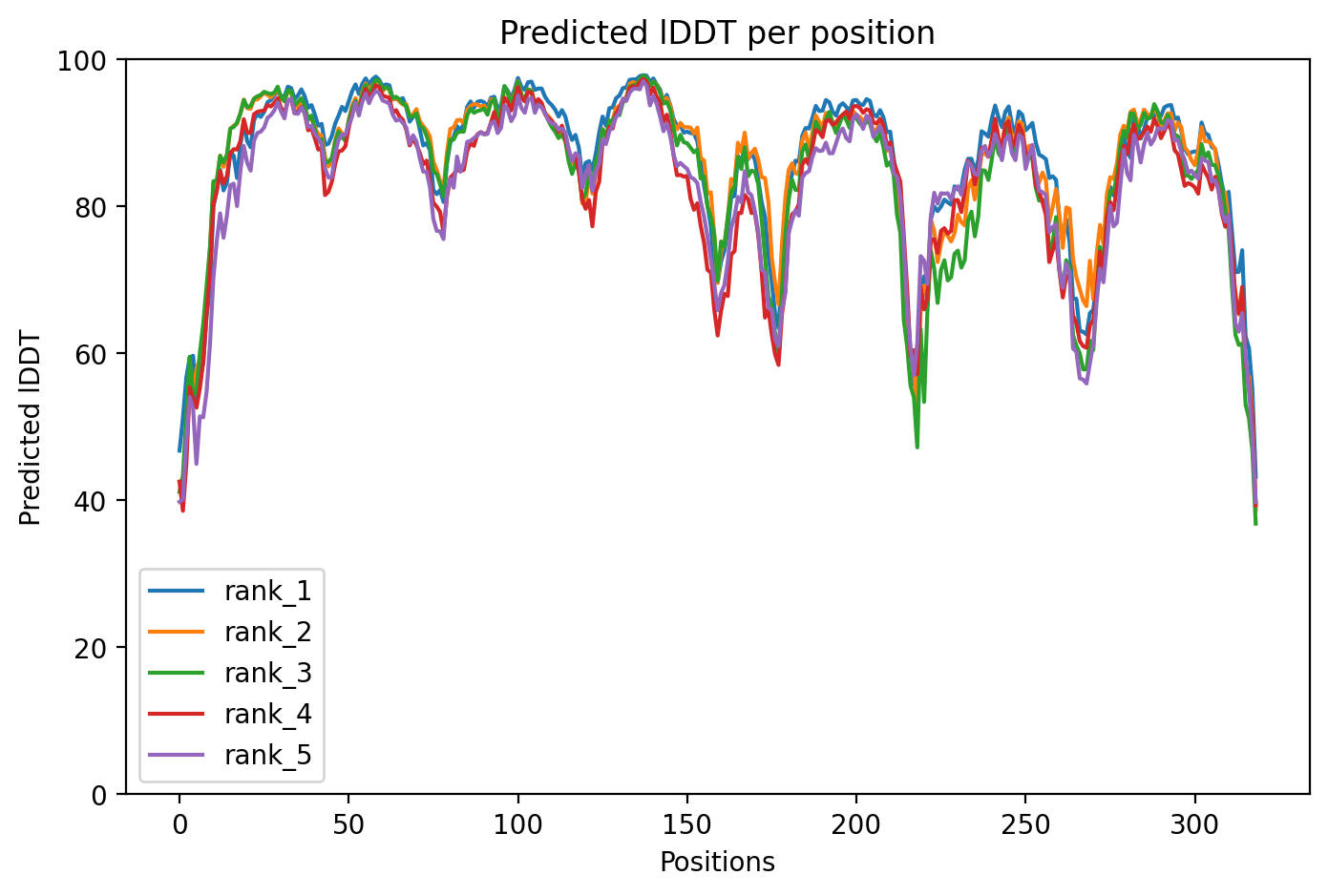

Supplement: Supplementary Data Sheet 1 — G. lateralis ESG transcriptome data set. [file DataSheet_1.zip › Supplementary Data/SuppData6/Gl-A24b1/af319_plddt.png]

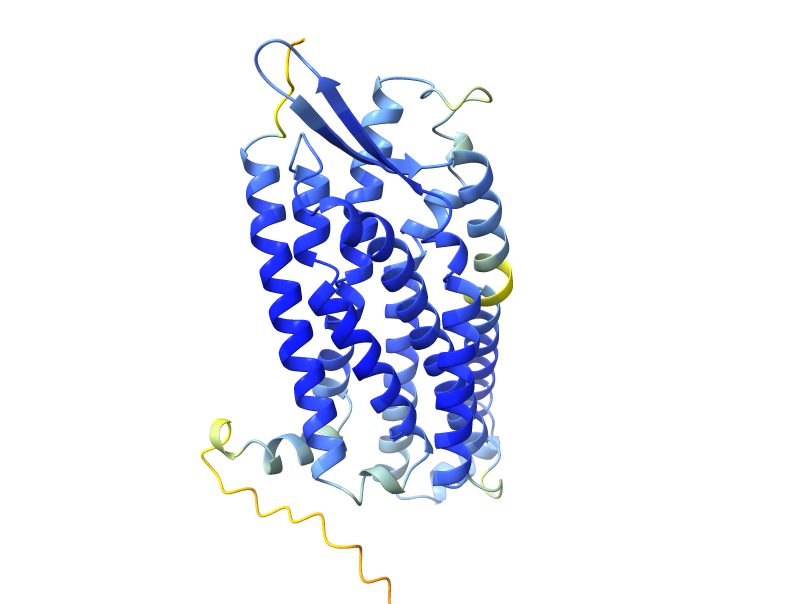

Supplement: Supplementary Data Sheet 1 — G. lateralis ESG transcriptome data set. [file DataSheet_1.zip › Supplementary Data/SuppData6/Gl-A34a2/A34a2.png]

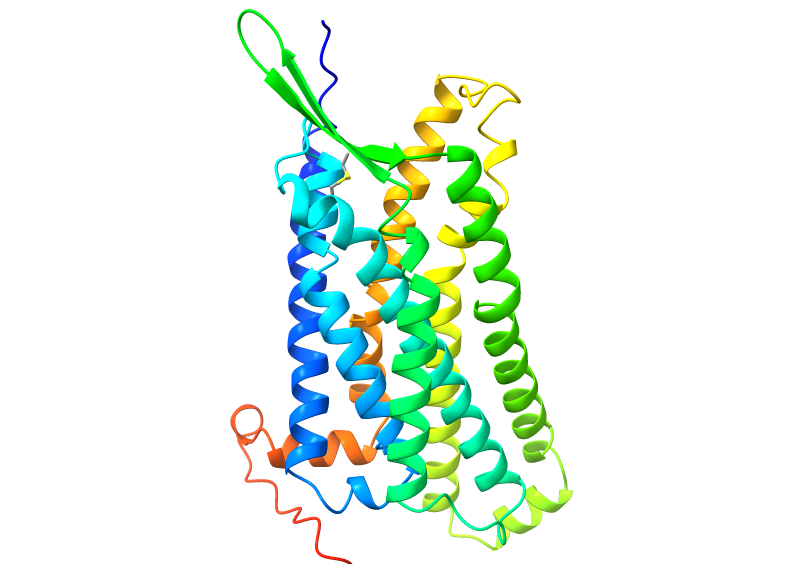

Supplement: Supplementary Data Sheet 1 — G. lateralis ESG transcriptome data set. [file DataSheet_1.zip › Supplementary Data/SuppData6/Gl-A34a2/A34a2-rainbow.png]

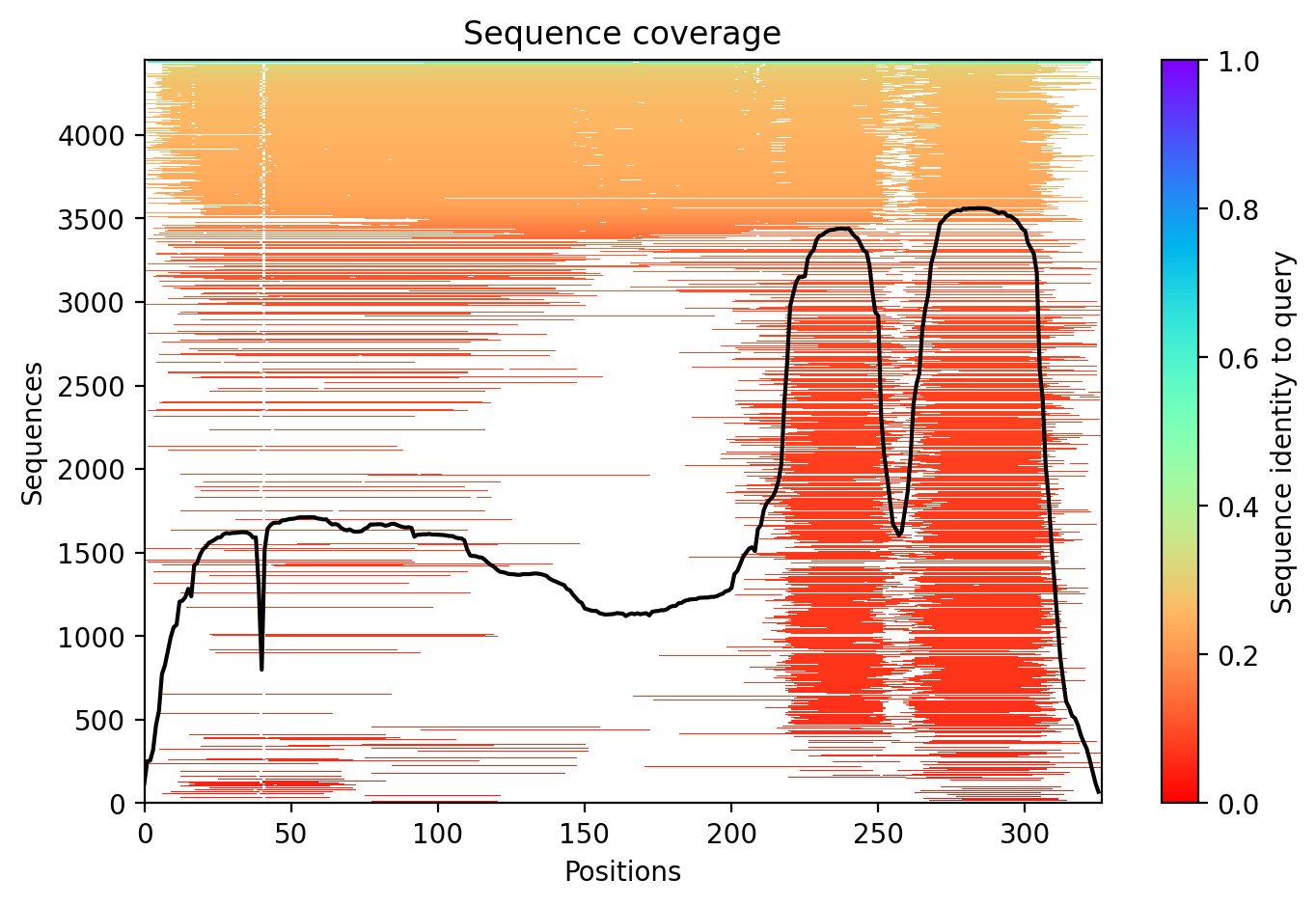

Supplement: Supplementary Data Sheet 1 — G. lateralis ESG transcriptome data set. [file DataSheet_1.zip › Supplementary Data/SuppData6/Gl-A34a2/af326_coverage.png]

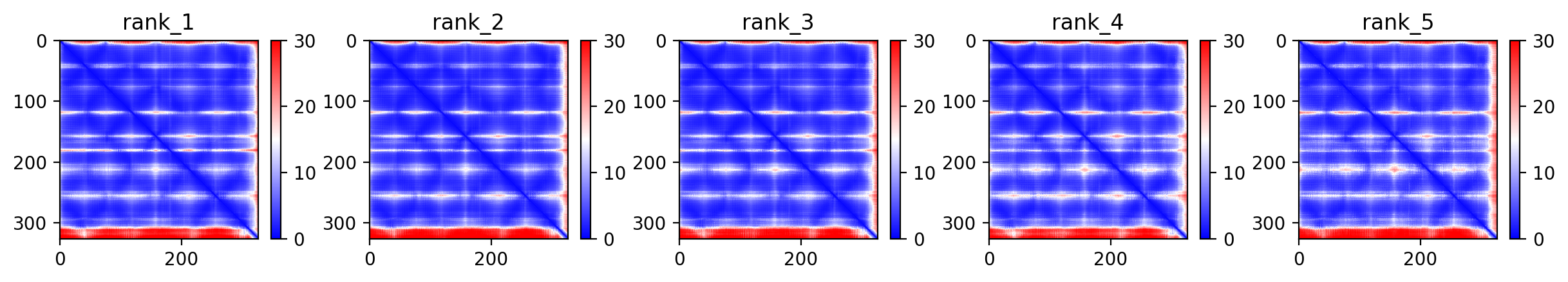

Supplement: Supplementary Data Sheet 1 — G. lateralis ESG transcriptome data set. [file DataSheet_1.zip › Supplementary Data/SuppData6/Gl-A34a2/af326_pae.png]

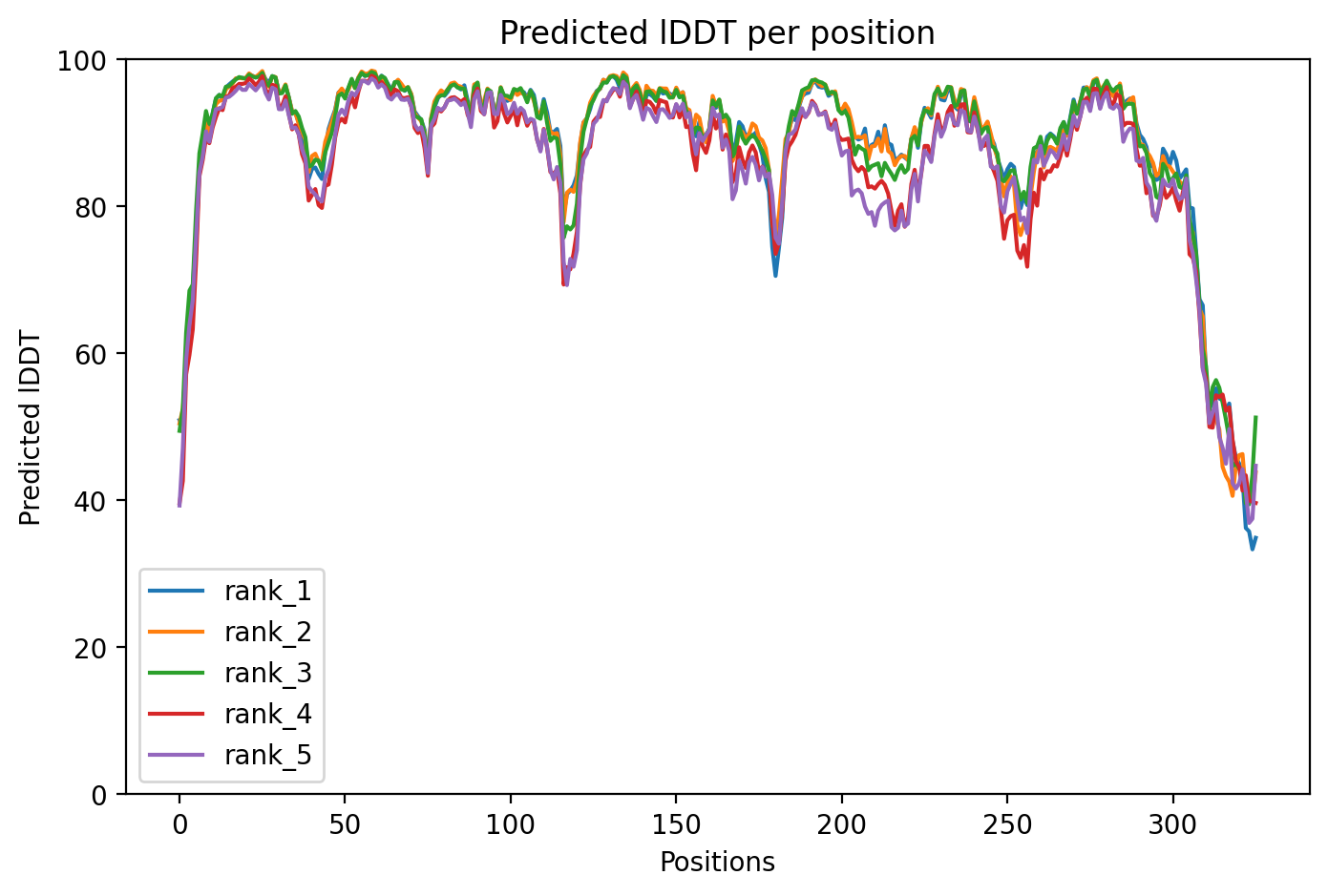

Supplement: Supplementary Data Sheet 1 — G. lateralis ESG transcriptome data set. [file DataSheet_1.zip › Supplementary Data/SuppData6/Gl-A34a2/af326_plddt.png]

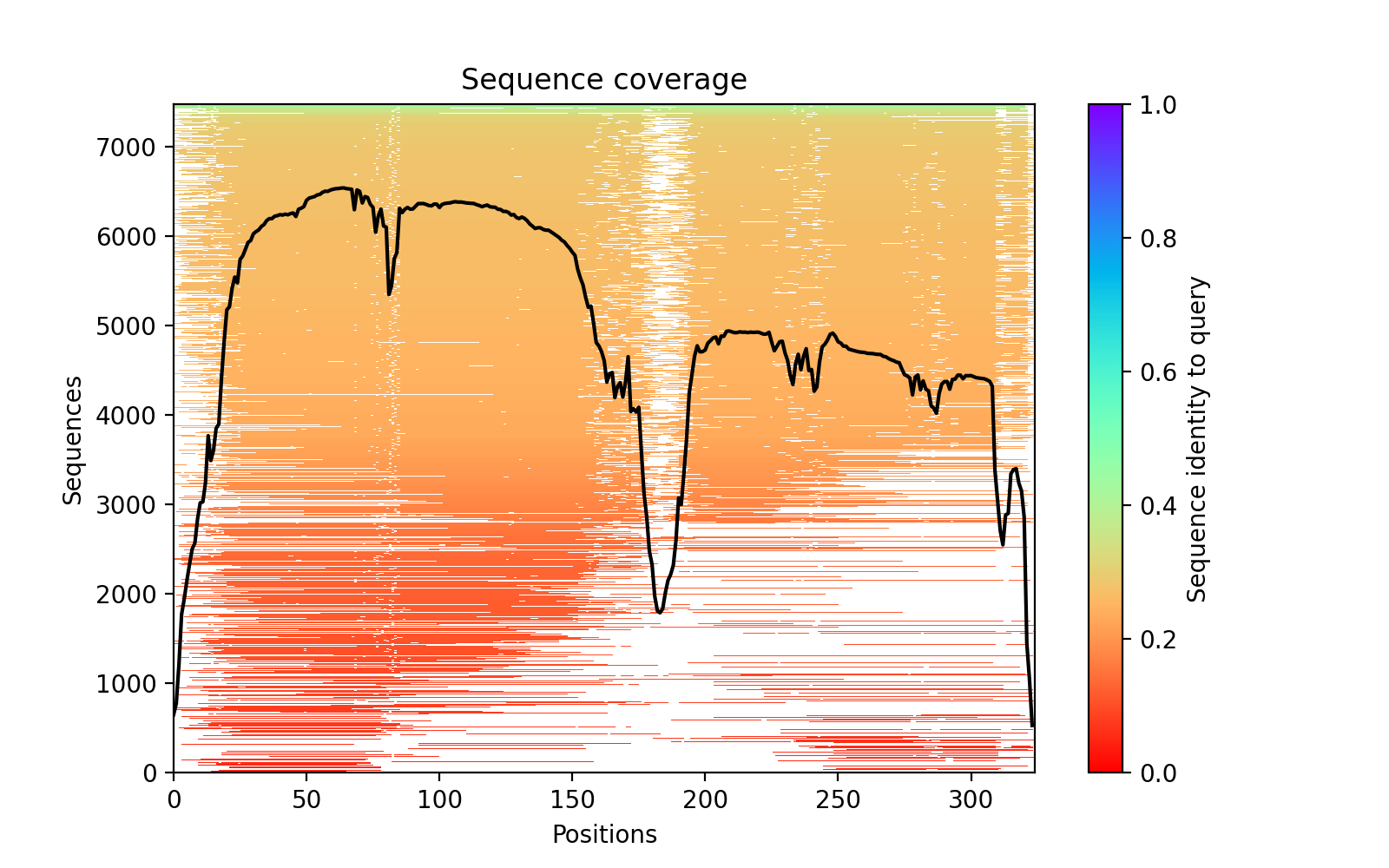

Supplement: Supplementary Data Sheet 1 — G. lateralis ESG transcriptome data set. [file DataSheet_1.zip › Supplementary Data/SuppData6/Gl-A34b1/af324_coverage.png]

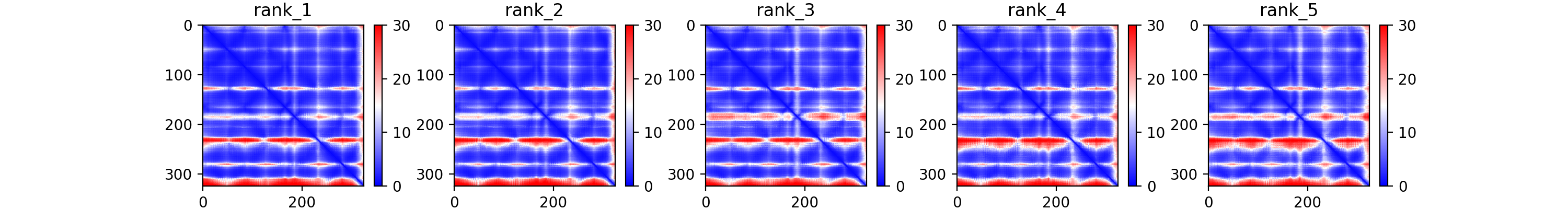

Supplement: Supplementary Data Sheet 1 — G. lateralis ESG transcriptome data set. [file DataSheet_1.zip › Supplementary Data/SuppData6/Gl-A34b1/af324_PAE.png]

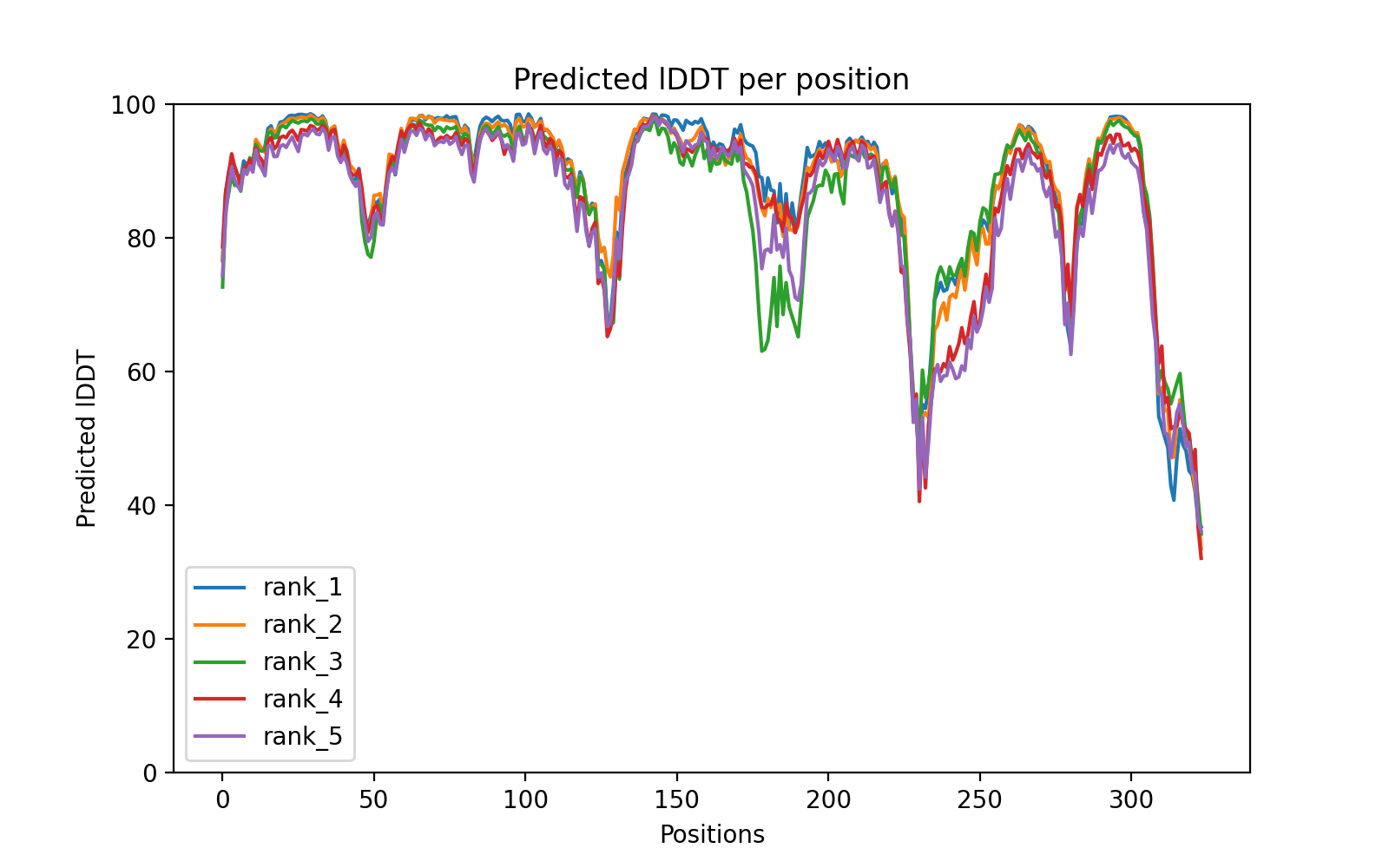

Supplement: Supplementary Data Sheet 1 — G. lateralis ESG transcriptome data set. [file DataSheet_1.zip › Supplementary Data/SuppData6/Gl-A34b1/af324_plddt.png]

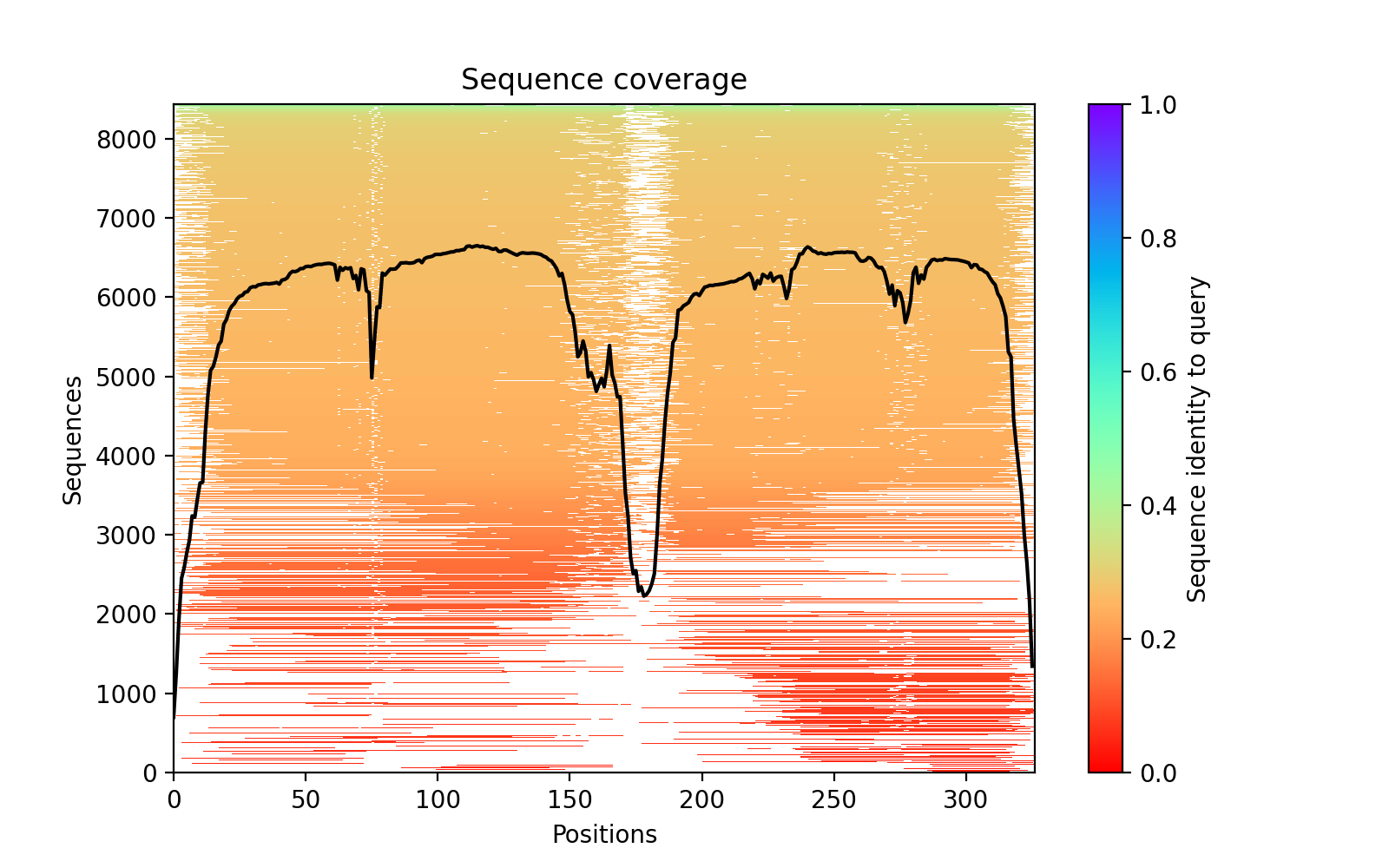

Supplement: Supplementary Data Sheet 1 — G. lateralis ESG transcriptome data set. [file DataSheet_1.zip › Supplementary Data/SuppData6/Gl-A34b2/af326_coverage.png]

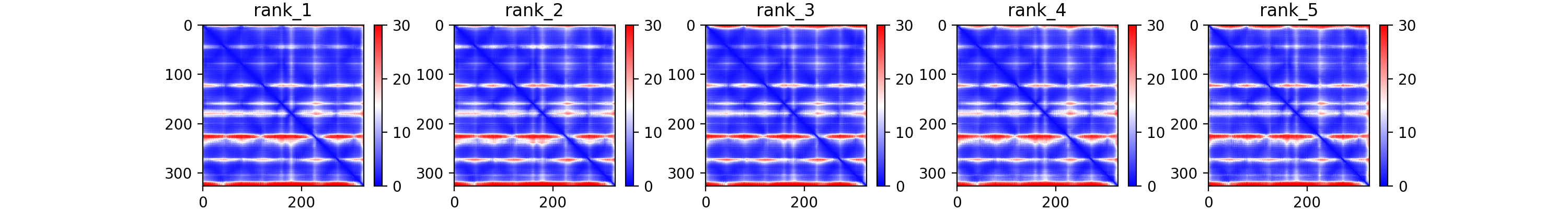

Supplement: Supplementary Data Sheet 1 — G. lateralis ESG transcriptome data set. [file DataSheet_1.zip › Supplementary Data/SuppData6/Gl-A34b2/af326_PAE.png]

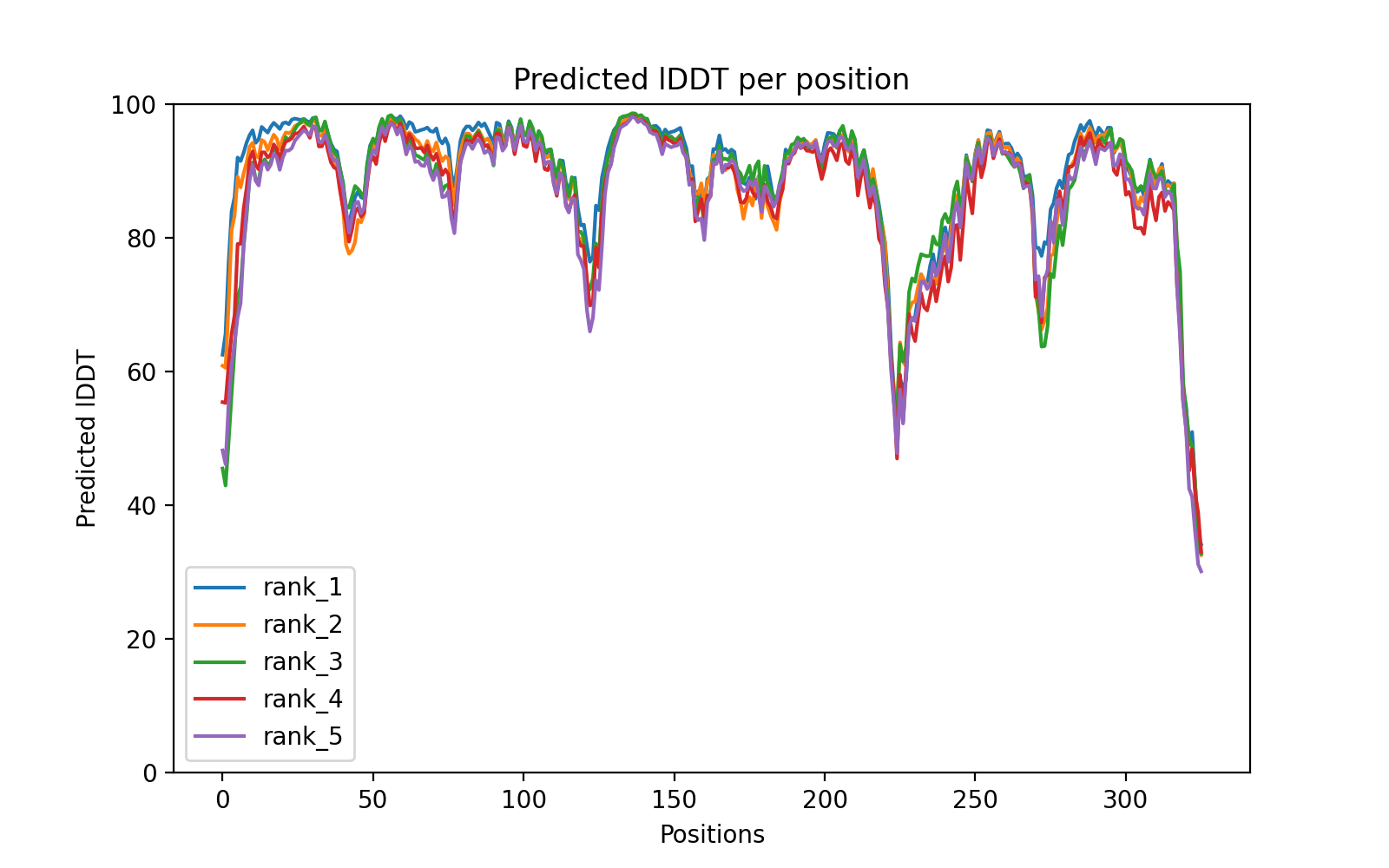

Supplement: Supplementary Data Sheet 1 — G. lateralis ESG transcriptome data set. [file DataSheet_1.zip › Supplementary Data/SuppData6/Gl-A34b2/af326_plddt.png]

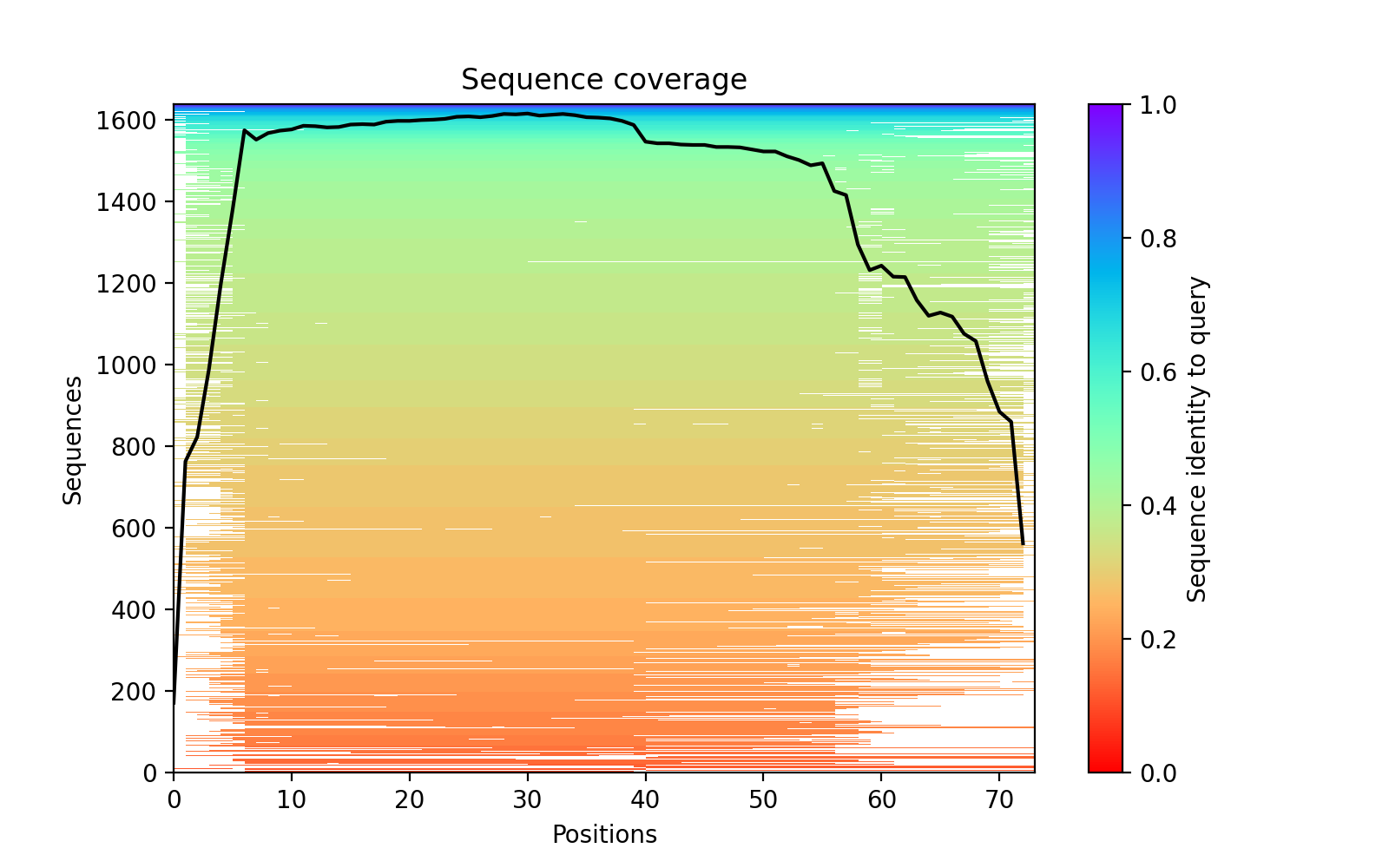

Supplement: Supplementary Data Sheet 1 — G. lateralis ESG transcriptome data set. [file DataSheet_1.zip › Supplementary Data/SuppData6/Gl-CHH/af73_coverage.png]

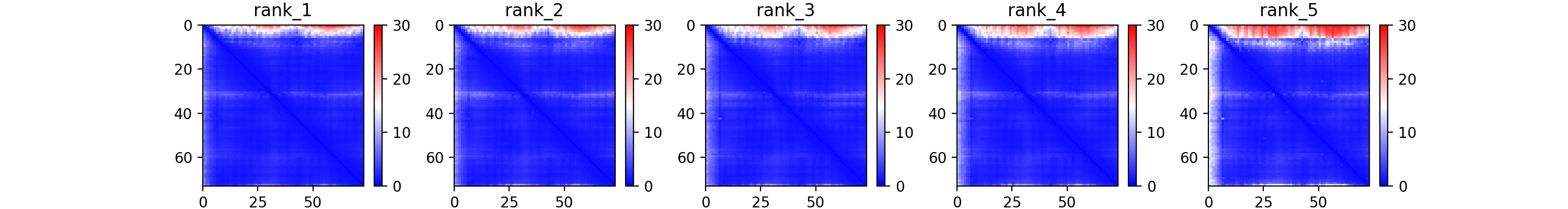

Supplement: Supplementary Data Sheet 1 — G. lateralis ESG transcriptome data set. [file DataSheet_1.zip › Supplementary Data/SuppData6/Gl-CHH/af73_PAE.png]

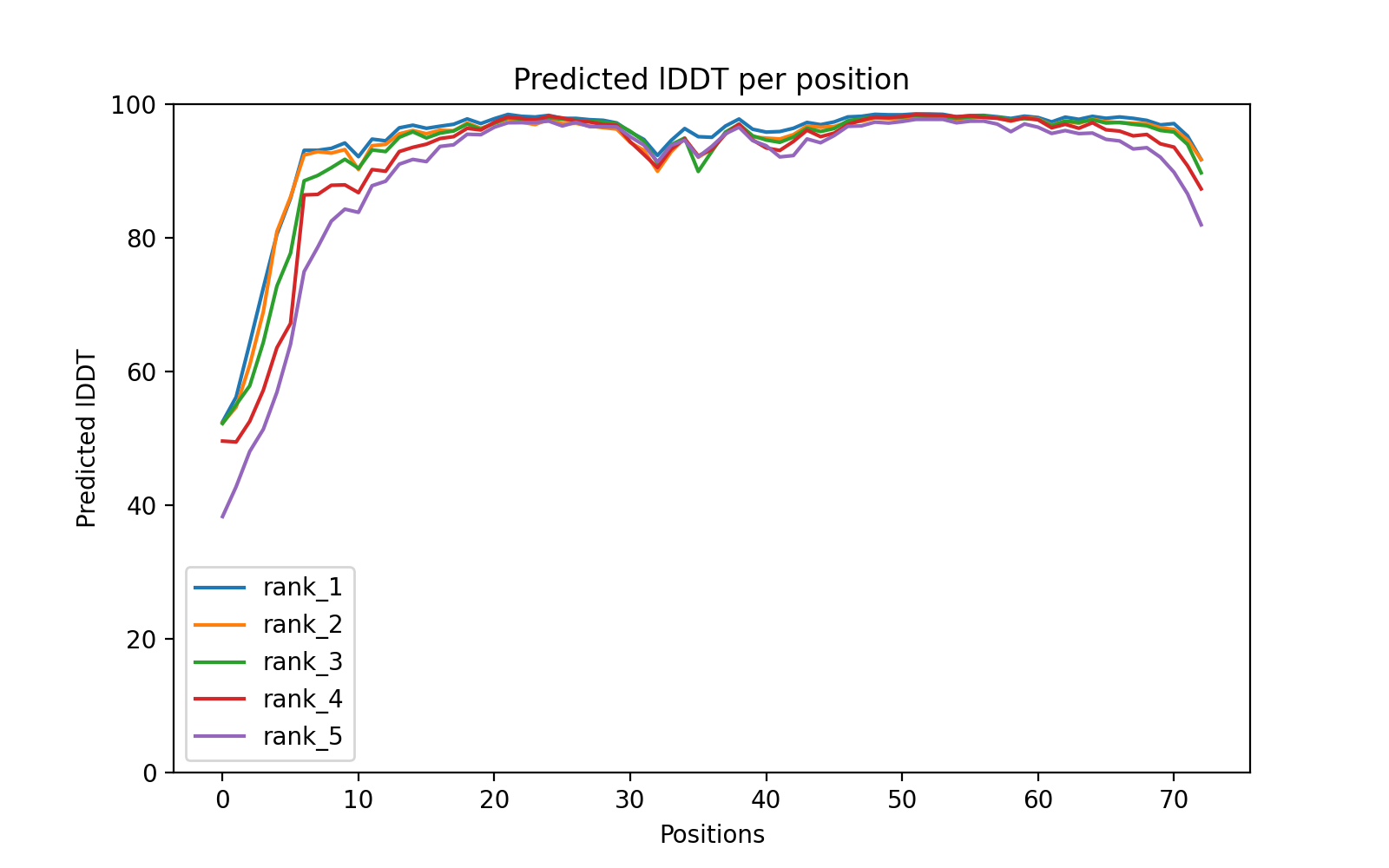

Supplement: Supplementary Data Sheet 1 — G. lateralis ESG transcriptome data set. [file DataSheet_1.zip › Supplementary Data/SuppData6/Gl-CHH/af73_plddt.png]

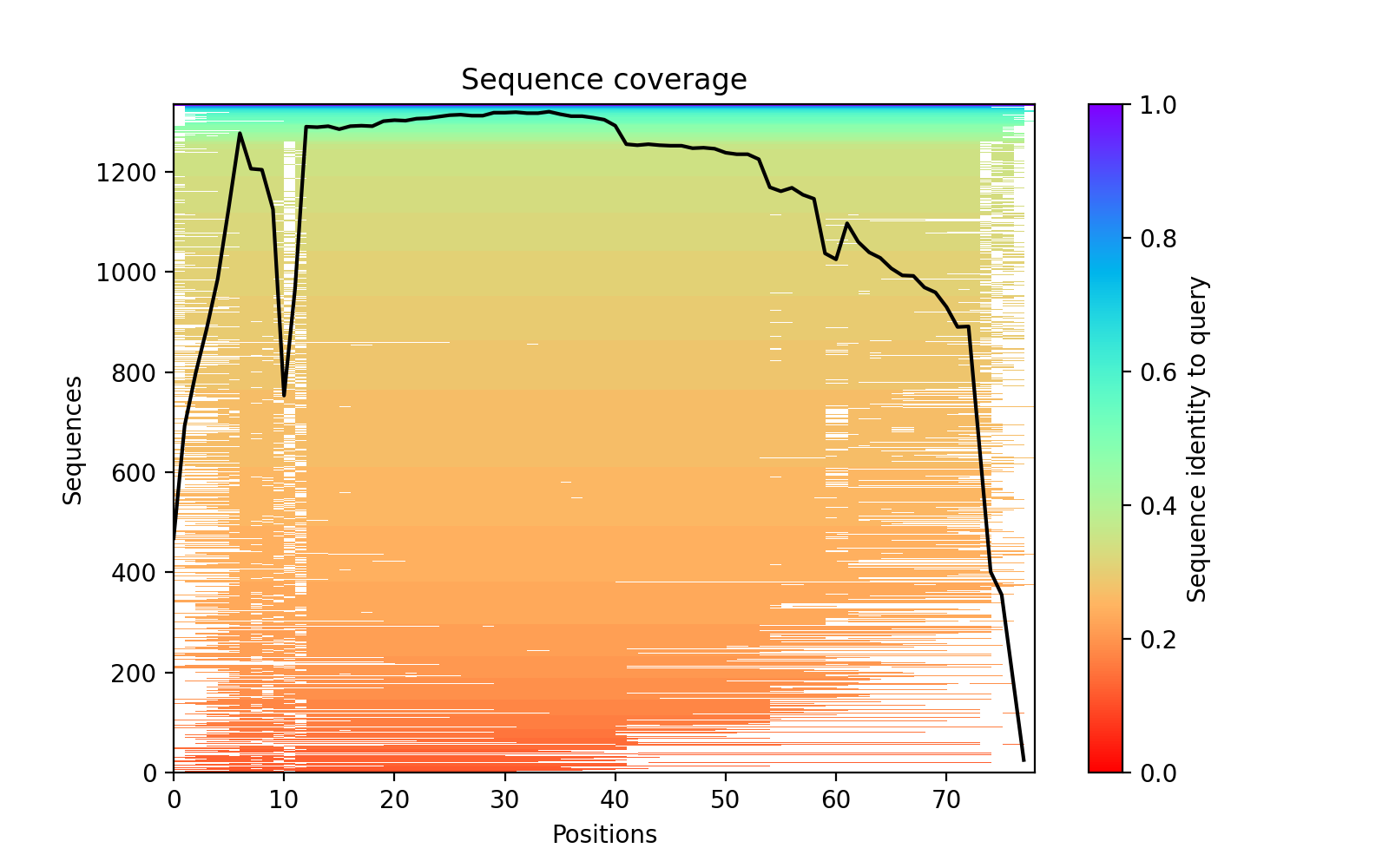

Supplement: Supplementary Data Sheet 1 — G. lateralis ESG transcriptome data set. [file DataSheet_1.zip › Supplementary Data/SuppData6/Gl-MIH/af78_coverage.png]

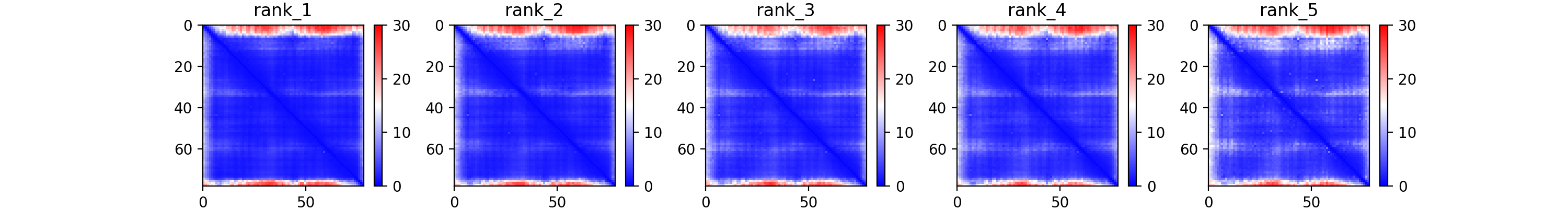

Supplement: Supplementary Data Sheet 1 — G. lateralis ESG transcriptome data set. [file DataSheet_1.zip › Supplementary Data/SuppData6/Gl-MIH/af78_PAE.png]

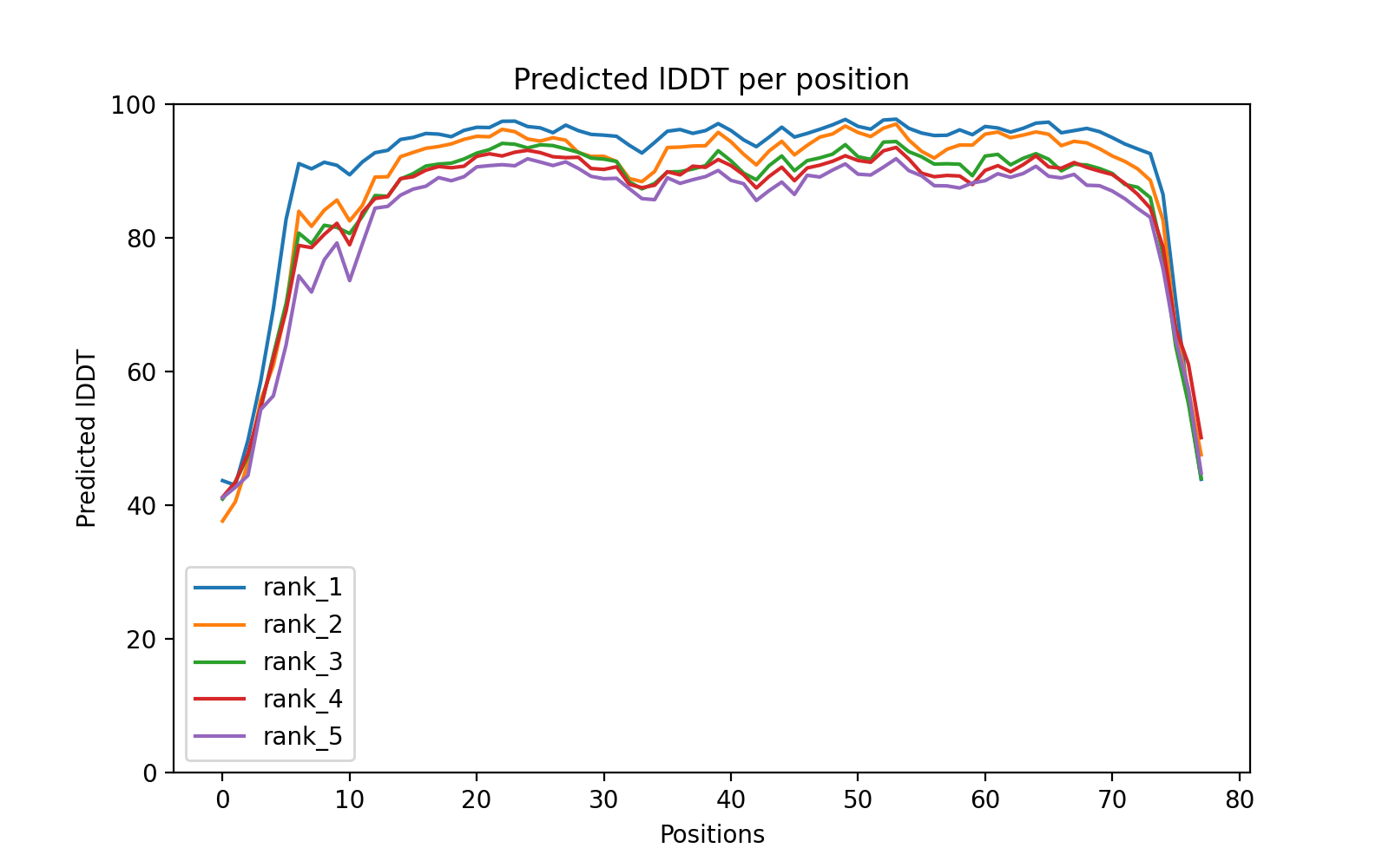

Supplement: Supplementary Data Sheet 1 — G. lateralis ESG transcriptome data set. [file DataSheet_1.zip › Supplementary Data/SuppData6/Gl-MIH/af78_plddt.png]
